# UK consensus guidelines for the delivery of unexpected news in obstetric ultrasound: The ASCKS framework

**DOI:** 10.1177/1742271X20935911

**Published:** 2020-08-04

**Authors:** Judith Johnson, Jane Arezina, Liz Tomlin, Siobhan Alt, Jon Arnold, Sarah Bailey, Hannah Beety, Ruth Bender-Atik, Louise Bryant, Jen Coates, Sam Collinge, Jo Fishburn, Jane Fisher, Jan Fowler, Tracey Glanville, Julian Hallett, Ailith Harley-Roberts, Gill Harrison, Karen Horwood, Catriona Hynes, Lindsay Kimm, Alison McGuinness, Lucy Potter, Liane Powell, Janelle Ramsay, Pieta Shakes, Roxanne Sicklen, Alexander Sims, Tomasina Stacey, Anushka Sumra, Samantha Thomas, Karen Todd, Jacquie Torrington, Rebecca Trueman, Lorraine Walsh, Katherine Watkins, Gill Yaz, Natasha K Hardicre

**Affiliations:** 1 School of Psychology, University of Leeds, Leeds, UK; 2 Yorkshire Quality and Safety Research Group, Bradford Institute for Health Research, Bradford Teaching Hospitals NHS Foundation Trust, Bradford Royal Infirmary, Bradford, UK; 3 University of New South Wales, Sydney, NSW, Australia; 4 Specialist Science Education Department (SSED), Leeds Institute of Cardiovascular and Metabolic Medicine, School of Medicine, Worsley Building, University of Leeds, Leeds, UK; 5 Bradford Teaching Hospitals NHS Foundation Trust, Bradford Royal Infirmary, Bradford, UK; 6 NHS Fetal Anomaly Screening Programme, Public Health England, London, UK; 7 Tiny Tickers, Leeds, UK; 8 University Hospitals Southampton NHS Foundation Trust, Southampton, UK; 9 Nest Independent Midwifery, Kirkstall, Leeds, UK; 10 The Miscarriage Association, Wakefield, UK; 11 Division of Psychological & Social Medicine, Leeds Institute of Health Sciences, University of Leeds, Leeds, UK; 12 SANDS (Stillbirth and Neonatal Death Society), London, UK; 13 University Hospitals Coventry and Warwickshire NHS Trust, Coventry, UK; 14 Northumbria Healthcare NHS Foundation Trust, North Tyneside General Hospital, North Shields, UK; 15 ARC (Antenatal Results and Choices), London, UK; 16 SOFT UK (Support Organisation for Trisomy 13/18), West Midlands, UK; 17 Leeds Teaching Hospitals Trust, Department of Fetal and Maternal Medicine, Leeds General Infirmary, Leeds, UK; 18 The Down’s Syndrome Association, London, UK; 19 Sunshine & Smiles – Leeds Down Syndrome Network, Leeds, UK; 20 Society and College of Radiographers, London, UK; 21 Independent Lay Expert, Leeds, West Yorkshire, UK; 22 College of Health, Wellbeing and Life Sciences, Sheffield Hallam University, Sheffield, UK; 23 Public Health England (PHE) Screening, London, UK; 24 Mid Yorkshire Hospitals NHS Trust, West Yorkshire, UK; 25 MVP (Maternity Voices Partnership), Leeds, UK; 26 Women’s Health Matters, Leeds, UK; 27 Independent Lay Expert, Huddersfield, West Yorkshire, UK; 28 School of Health and Human Sciences, Southern Cross University, New South Wales, Australia; 29 Royal Free London NHS Foundation Trust, Barnet Hospital, Barnet, UK; 30 St Mary’s Hospital, Manchester University Foundation Trust, Manchester, UK; 31 University of Huddersfield, Huddersfield, UK; 32 Calderdale and Huddersfield Foundation Trust, University of Huddersfield, Huddersfield, UK; 33 School of Health Sciences, Birmingham City University, Edgbaston, UK; 34 Sydney School of Health Sciences, Faculty of Medicine and Health, University of Sydney, New South Wales, Australia; 35 Department of Health and Social Care, London, UK; 36 Division of Midwifery and Radiography, School of Health Sciences, City, University of London, London, UK; 37 Birmingham Children’s Hospital, Birmingham, UK; 38 Leeds Teaching Hospital Trust, Leeds General Infirmary, Antenatal Clinic, Leeds, UK; 39 SHINE (Spina Bifida, Hydrocephalus, Information, Networking, Equality), Peterborough, UK

**Keywords:** Ultrasound, sonography, communication, news delivery, guidelines, training

## Abstract

**Background:**

Studies indicate there is a need to improve the delivery of unexpected news via obstetric ultrasound, but there have been few advances in this area. One factor preventing improvement has been a lack of consensus regarding the appropriate phrases and behaviours which sonographers and ultrasound practitioners should use in these situations.

**Aims:**

To develop consensus guidelines for unexpected news delivery in Early Pregnancy Unit and Fetal Anomaly Screening Programme NHS settings.

**Methods:**

A workshop was conducted to identify priorities and reach consensus on areas of contention. Contributors included interdisciplinary healthcare professionals, policy experts, representatives from third-sector organisations, lay experts and academic researchers (*n* = 28). Written and verbal feedback was used to draft initial guidance which was then circulated amongst the wider writing group (*n* = 39). Revisions were undertaken until consensus was reached.

**Results:**

Consensus guidelines were developed outlining the behaviours and phrases which should be used during scans where unexpected findings are identified. Specific recommendations included that: honest and clear communication should be prioritised, even with uncertain findings; technical terms should be used, but these should be written down together with their lay interpretations; unless expectant parents use other terminology (e.g. ‘foetus’), the term ‘baby’ should be used as a default, even in early pregnancy; at the initial news disclosure, communication should focus on information provision. Expectant parents should not be asked to make decisions during the scan.

**Conclusions:**

These recommendations can be used to develop and improve news delivery interventions in obstetric ultrasound settings. The full guidelines can be accessed online as supplemental material and at https://doi.org/10.5518/100/24.

## Introduction

Delivering unexpected news in obstetric ultrasound is challenging for healthcare professionals and can be a distressing experience for expectant parents.^1–4^ News delivery practices in obstetric settings vary internationally and the UK is one of few countries where sonographers deliver information about unexpected findings as standard.^5^ In other countries such as the US and Australia, policies differ according to the healthcare discipline of the ultrasound practitioner, their relationship with the referring clinician and the type of complication which has been identified.^6–8^

Studies suggest that a policy of immediate disclosure is consistent with patient preferences,^4,9^ but that this practice can be stressful for healthcare professionals because, due to the immediate nature of the findings, they have no time to prepare before communicating with expectant parents.^2,4^ This situation is not acknowledged in current news delivery frameworks, which are based in oncology settings and presume that clinicians will have time to plan news disclosures before meeting with patients.^10,11^ Furthermore, in obstetric ultrasound settings, unexpected findings may be uncertain^12,13^ and professionals may be working under restrictive time constraints.^14,15^ It is perhaps unsurprising, then, that expectant parents continue to report negative experiences of care at this time, such as delays in news delivery^3^ and the use of insensitive language by staff.^16^

In the UK, there is no mandatory or standardised pre-qualification training for sonographers and ultrasound practitioners in communication. Most trainees receive some teaching on this topic as part of their course and describe learning skills for unexpected news delivery while on placement.^1^ Post-qualifying, a majority of sonographers seek continued professional development training in this area.^17^ However, one unresolved challenge for sonographers and ultrasound practitioners relates to the specific words, phrases and behaviours that they should use in unexpected news delivery situations.^1^ Whilst principles such as compassion and sensitivity may be widely recognised,^13,18^ it is not possible to communicate in principles and parents who receive unexpected news often recall the exact words their ultrasound practitioner used.^16,19^ One study found that trainee sonographers carefully observed the language and behaviours of qualified staff in practice and selected those they believed reflected best practice.^1^ However, this approach has limitations. First, it is demanding of energy and cognitive resources, as each trainee is effectively tasked with constructing their own ‘best practice’ guidelines. Second, if the qualified sonographers in a department offering a placement are not using phrases which reflect best practice, trainees may learn these phrases and poor practice will be replicated. Third, there is a wide array of potential news delivery scenarios that a sonographer may encounter during their career; it is unlikely they will have the opportunity to observe each of these during their training placements.

The lack of consensus regarding appropriate words, phrases and behaviours in news delivery also has negative implications for practice improvement. Until recommendations are clear and transparent, it is not possible to discuss, test and refine these. The current project addressed this issue by generating detailed consensus guidelines regarding best practice for news delivery in obstetric ultrasound. It aimed to create recommendations which healthcare professionals, policy experts, expectant parent representative groups, lay experts and academics agreed were appropriate. The broader goal was to make recommendations for news delivery in obstetric ultrasound explicit, such that these can be reviewed, discussed and tested by subsequent research, to enable ongoing improvement in future. The guidance focused on UK Early Pregnancy Unit (EPU) and Fetal Anomaly Screening Programme (FASP) settings, where unexpected news is delivered by sonographers and ultrasound practitioners as standard.

## Methods

### Design and procedure

A workshop was hosted to discuss news delivery in obstetric ultrasound. This included group discussions and individual exercises. Priorities were identified and areas of contention were discussed to enable problem solving. Case studies were used to raise pertinent issues arising in different news delivery scenarios. Feedback was audio-recorded and participants provided written feedback during individual exercises. Following the workshop, the initial guidance document was circulated to attendees and to the wider writing group. Comments were used to revise the document, and the revised document was circulated to all contributors on two further occasions until consensus was reached.

### Participants

The workshop was attended by 28 participants from across the UK and included 6 sonographers, 4 midwives, 1 obstetrician, 4 academics, 2 lay experts, 8 representatives from third-sector organisations and 3 policy experts. The wider writing group included 39 participants in total (10 sonographers, 6 midwives, 1 obstetrician, 5 academics, 3 lay experts, 10 representatives from third-sector organisations and 4 policy experts, including those who attended on the day).

### Ethical considerations

Ethical approvals were not sought as the project aimed to derive consensus guidelines; participants were invited to be recognised as authors, and as such, standard confidentiality policies which would be applied to research projects were not utilised. However, all participants were asked to agree to a policy of confidentiality regarding the workshop discussions. Audio recordings and written feedback were stored securely until the guidelines had been drafted and circulated to the writing group. After completion, audio recordings and written feedback were destroyed.

## Results

The full document outlining detailed consensus guidelines is included in Appendix 1. Key phrases, summarised here, are provided under the main topics of ‘setting up the scan’, ‘during the scan’, ‘phrases to communicate pregnancy loss’, ‘phrases to communicate foetal conditions’ and ‘ending the scan’. Key principles contained within the guidelines are summarised in Table 1. These are: Avoid assumptions; Set up the scan; Clear, honest information; Kindness; Self-care; they provide the letters from which the framework name is derived (ASCKS).

**Table 1.** Key principles for news delivery in obstetric ultrasound.

Key principles	Things to consider
**Avoid assumptions**	Remain aware that people may not react in the way you might expect them to. Use neutral terms (e.g. ‘unexpected’ rather than ‘abnormal’) and make no assumptions.
**Set up the scan**	Gathering information and setting expectations prior to all scans can facilitate better communication in those where unexpected findings are identified. Use the preamble prior to undertaking the scan to: **Introduce yourself** and your role **Check understanding** about the purpose of the scan **Assess feelings** about the pregnancy **Explain that you will be silent** at times during the scan **Check consent** for the scan and any screening **Explain when and how you will show them the monitor** In the first trimester, also **check their pregnancy dates** and **explain if you think an internal scan might be needed**
**Clear, honest information**	Providing expectant parents with clear, honest information can help them to understand and process the news. The following suggestions can support clear communication: **Put down the probe, turn and make eye contact** before verbally communicating the news Use **technical terms but also provide the ‘lay translation’** of what these mean **Communicate exactly what will happen next and why** – whether this is an internal scan or leaving the room to seek a second opinion Wherever possible, **offer written information and signpost** to relevant organisations which can offer further information and/or support
**Kindness**	Kindness and compassion are key to better news delivery. The following suggestions can support kindness in news delivery situations: Unless you hear expectant parents using other terminology (e.g. ‘foetus’), **use the term ‘baby’ as a default**, even in very early pregnancy **Understand the nature of shock**: it is a common phase where parents ‘make sense’ of the news they have been told. People struggle to assimilate new information during this time; **avoid asking them to immediately make decisions** around pregnancy management. **Express regret**: Where a condition has been found**, hold the ‘I’m sorry’** until after you have delivered the initial news and only if this feels appropriate. In doing this, you are expressing regret about their distress, not the finding itself.
**Self-care**	Remember that delivering unexpected news via ultrasound is uniquely demanding. Have compassion for yourself, as well as those you are scanning. Explore coping strategies which are useful to you and make time to care for yourself.

## Setting up the scan

The pre-scan discussion can help build rapport with expectant parents and help set their expectations. This can facilitate a better news delivery experience in those scans in which the need arises. This discussion should include the following elements:

### Introductions

Many expectant parents are not clear on what the role of a sonographer involves; a simple introduction including the sonographer’s name, outlining their role and explaining the purpose of the scan can help set expectant parents’ expectations and put them at ease.

### Checking understanding

The woman’s awareness and understanding of the purpose of the scan should be checked using the ‘Teach Back’ technique.^20^ This has two principles. First, the healthcare professional puts the spotlight on themselves or the process rather than the patient, to reduce the likelihood that patients will perceive this as a test or criticism. Second, the healthcare professional asks the patient to tell them what they know about the information they have just been given. For example, ‘To be sure that I have explained everything clearly, could you explain to me what you understand the purpose of the scan to be?’

### Assessing feelings about the pregnancy

Asking a question about how the woman feels about the pregnancy can elicit information about concerning pregnancy symptoms or a difficult obstetric history which can inform subsequent communication if unexpected findings are identified. For example, ‘Before I start the scan, I like to check in with women to see how they are feeling. So, can I ask, how are you feeling about the pregnancy?’

### Checking consent

Consent is an iterative process; it cannot be assumed that decisions made in a previous appointment will still be the same at a subsequent appointment. As such, it is important that women are informed about the purpose of the scan and that consent is gained before each examination is conducted. For example, after clarifying the purpose of the scan, the sonographer could ask, ‘Before I continue, I need to check – do you consent to proceeding with the scan?’

### Explaining the silence

Many expectant parents have reported finding the silence during scanning anxiety provoking. It should be explained at the start of the scan that there will be a period of silence and how long this will last for to help reduce this anxiety. Any reasons that may increase the length of the silence, such as being new to scanning, should also be explained.

### Explaining how and when the monitor image will be shared

Sonographers and ultrasound practitioners should consider whether and how they will show expectant parents images on the screen, and explain this to them at the start of the scan. It is important that the screen is not initially shared with expectant parents and then turned away in the event of an unexpected finding.

### First trimester considerations

In first trimester scans, it is important to check the woman’s confidence in her dates prior to the scan. This question should not be asked part way through a scan when unexpected findings have been identified. If an internal scan might be needed, this should also be explained at the start of the scan. This should be offered as an option, recognising that some women would prefer to wait for a subsequent appointment rather than receive an internal scan.

### When expectant parents bring children

As part of standard protocol, many hospitals ask expectant parents not to bring children to the scan. However, children are still often brought to scans for various reasons. Due to the wide range of different potential situations, it is not possible to prescribe specific guidance which can be applied to every situation. Instead, an individual strategy is needed for each situation, dependent upon what each sonographer thinks is appropriate. Sonographers can draw on three principles to help guide their decision-making in these situations:

1. Choice: where possible, parents should be provided with options about how to proceed with the scan, as this can help them to feel more in control of the situation.
2. Avoiding delays: once unexpected findings have been identified, it is important this is communicated as soon as possible.
3. Duty of care: the underpinning principle must remain to provide the best care possible; providing compassionate, patient-centred care which is tailored to each situation and which is sensitive to the needs of both the parents and children who are attending the scan.

## During the scan

While the delivery of unexpected news will vary significantly between different situations, there are some key principles which are relevant for all scans:

### Behaviours

When delivering unexpected news identified by transabdominal scan, sonographers and ultrasound practitioners should put down the probe first, before turning to face expectant parents and making eye contact. This provides a behavioural cue and puts the professional in a physical stance which communicates engagement and care. If conducting an internal scan, allow the woman to choose whether she would prefer to receive the news while the probe is in-situ or to get dressed first. For example ‘I’ve seen something that I’m not expecting to see at this stage of pregnancy. Would you prefer to talk about this now while I can show you the screen, or would you prefer to get dressed, and then talk?’

### Neutrality

Unexpected news in ultrasound is often not objectively ‘good’ or ‘bad’. How it is received will depend on the situation, experiences and views of the expectant parents who are receiving the news. As such, it is important that phrases such as ‘bad news’ and ‘good news’ or ‘normal’ and ‘abnormal’ are avoided when describing findings. Instead, neutral phrases such as ‘expected’ and ‘unexpected’ should be prioritised. It is also important that the term ‘chance’ is used rather than ‘risk’ when conveying likelihoods of possible outcomes. To note here, while the term ‘unexpected news’ is usually preferable to non-neutral terms (e.g. ‘bad news’), it should be considered that for a minority of parents, this news may not be unexpected due to their previous experiences. In these situations, it can be useful to acknowledge this while maintaining neutral language, for example ‘The scan confirms what the previous test suggested’.

### ‘Baby’

The default term when communicating with expectant parents should be ‘baby’ rather than ‘pregnancy’ or ‘foetus’, as this is preferred by most expectant parents and is used in the literature provided by Public Health England.^21^ However, the language used by each woman should be considered. If she or her partner use another term, sonographers and ultrasound practitioners should also adopt this term during the scan.

### Understand the nature of shock

When expectant parents receive unexpected news via ultrasound, they may go into shock.^19^ The degree of shock will vary between people but the underlying process is similar: they are assimilating new information into their broader understanding of themselves, their life and their world.^22^ A consistent observation is that expectant parents may struggle to recall information which is provided to them during this period. It is important to understand that expectant parents will not want to be immediately presented with options and asked to make decisions when they are experiencing shock.

### Expressing regret

It is appropriate to open the news by saying ‘I’m sorry to tell you that….’ in situations where there has been any form of pregnancy or baby loss, or where a life-limiting condition has been identified. In situations where a condition is identified which may not be life-limiting or is clearly not life-limiting, it is better to ‘hold’ the sorry. That is, the news should be delivered using a phrase such as ‘I have found that …’. If expectant parents then express sadness, anger, shock or distress, this can then be followed with a phrase such as, ‘I’m sorry, I know this is not what you were expecting to hear’. The underlying principle is that while the news of the baby’s condition might be unexpected, healthcare professionals should not try to predict how this news will be received by the expectant parents. By ‘holding’ the ‘sorry’ until after their initial reaction, the apology relates to the distress they are experiencing, rather than the finding itself.

### Technical/translation

When communicating with expectant parents, it is important that lay terms are used to describe the finding, to help them understand what this means for them. However, it is also important that they are provided with the technical term. This is because most expectant parents will search the internet for information about the results of the ultrasound scan. Without the correct technical term, it is more likely that they will come across inappropriate or misleading information. The technical term should be provided to expectant parents in written or printed format for them to take away.

### Reaffirm emotions

Reaffirming a person’s emotions can help them to feel heard and understood. This can be done by ‘naming’ the emotion they seem to be expressing, whether this is sadness, shock, disbelief or anger and expressing regret for this, for example ‘I’m so sorry, I know this is really sad news and it must be hard to take in’. Where appropriate, reaffirming statements can be combined with ‘wish’ statements, such as ‘I wish I didn’t have to say this’, or ‘I wish things were different’.^23^

### Managing challenging issues

If a second opinion is needed, sonographers and ultrasound practitioners should be honest about the reason for this. Expectant parents should be advised how long they may be waiting and the second healthcare professional should be introduced when they come into the room. If the news is uncertain, this uncertainty should be communicated honestly to expectant parents. They should be provided with a balanced picture of the finding, which avoids catastrophising (giving only a ‘worst-case scenario’) or offering false reassurance (giving only a ‘best case scenario’).

## Phrases to communicate pregnancy loss

Terms which should be avoided when communicating news of a pregnancy loss, together with preferred alternatives, are outlined in Table 2.

**Table 2.** Terms which should be avoided when communicating news of pregnancy loss.

Terms to avoid altogether	Alternative terms	Example phrase
**Blighted ovum/anembryonic pregnancy**	A baby who died very early on	‘I can see a pregnancy sac in your womb, which is the place where the baby grows. Sadly though, I cannot see a baby in the pregnancy sac as it is likely that it died very early on before we could see it on the scan’
**Products/products of conception**	Tissue/pregnancy tissueRemains of the pregnancy	‘I can still see the tissue which was left in your uterus from when your baby was growing’ ‘There are still some remains of the pregnancy in your womb from when your baby was developing’
**Evacuation of retained products/vacuumed out/scraped**	Surgery to remove tissue from your womb	‘This is surgery to remove the tissue that was left in your womb from the developing baby’
**Non-viable**	Will not continue to develop	‘The baby will not continue to develop, and won’t survive’
**Incompetent cervix/ Cervical insufficiency**	Opening before it should	‘Your cervix is ‘dilating’ – which means that it is opening before it should’
**Abortion (or any variation of this, such as ‘threatened’, ‘missed’ or ‘incomplete’)**	Miscarriage	‘Unfortunately you have miscarried, but this is what we call a ‘missed miscarriage’, or a ‘delayed miscarriage’ which means that you are still carrying the baby but it has stopped developing’
**IUD/Intra-uterine death**	Baby has died	‘I’m afraid I can see that your baby’s heart has stopped beating [*pause*], and this means that your baby has died’

Some terms are unlikely to be hurtful or offensive, but may be confusing and need translating. Example terms and their translation are outlined in Table 3.

**Table 3.** Terms which should be translated when communicating news of pregnancy loss.

Technical term	Lay translation	Example phrase
**ERPC/surgical management of miscarriage**	Surgery to remove tissue from the womb	‘This is surgical management of miscarriage, which is the technical term to describe surgery to remove what was left in your womb from the developing baby’
**Gestational/yolk/pregnancy sac**	The place in which the baby grows	‘I’m looking at the pregnancy sac, which is the place where the baby grows’
**Haematoma**	An area of bleeding in or near the pregnancy sac	‘I can see there is an area of bleeding next to the pregnancy sac (the place where the baby grows). This is called a haematoma.’
**Foetal pole**	The first sign of a developing baby	‘I can see the first sign of a developing baby’

### Early loss prior to a heartbeat

While the pregnancy does not resemble a baby at this time, it is important to recognise that many expectant parents will not be aware of this and may be shocked and confused by the use of technical language referring to ‘gestation sacs’, and ‘foetal poles’. If they have talked about their pregnancy with others, they will likely have used common terms such as ‘pregnant’ and ‘baby’ to describe their experience. As such, these terms should be used by default, unless the expectant parents are using alternative terms themselves. Where a foetal pole is not visible (i.e. where the pregnancy is anembryonic), the term ‘first sign of a developing baby’ should not be used because if expectant parents ask to see the screen, this will not be visible. Instead it should be stated clearly that there is a pregnancy sac but a baby cannot be seen within the sac as it died whilst it was too small to be seen with ultrasound.

### Early loss following a heartbeat

This finding should be communicated in simple, clear language, explaining that the baby has ‘died’. Wish statements can be used if appropriate. The next steps (e.g. the health professional they will see next) should be explained before the end of the scan.

### Baby loss

While the term ‘I’m sorry’ is contentious in relation to some obstetric ultrasound findings, it is important that this is used in situations where ultrasound identifies a late pregnancy loss or stillbirth. Example phrases include ‘I am afraid it is unexpected news … ’, and ‘I’m sorry, I’m afraid I have found that … .’.

## Phrases to communicate foetal conditions

Terms which should be avoided when communicating news of foetal conditions, together with preferred alternatives, are outlined in Table 4.

**Table 4.** Terms which should be avoided when communicating news of a possible foetal condition.

Terms to avoid altogether	Alternative terms	Example phrase
Normal	Expected	‘This looks as we would expect at this point in the baby’s development’
Abnormal	Not as expected/unexpected	‘This is not what I would expect to see during this phase of baby’s development’
Disorder	Condition	‘This means the baby might have a condition called [*insert technical name of condition*]’
Incompatible with life	Will not be able to survive	‘Unfortunately, this means that the baby will not be able to survive’
Lemon-shaped	Narrows	‘The baby’s head narrows at this point, which may indicate something unexpected with baby’s development’
Banana-shaped	Curved	‘This part of the brain is curved, which may indicate something unexpected with baby’s development’

### Key principles

There is a wide range of potential foetal conditions which could be identified via obstetric ultrasound. However, the following principles apply to all findings:

- Value-laden language should be avoided. The alternative terms in Table 4 provide direction on how this can be achieved.
- Sonographers and ultrasound practitioners should not try to diagnose beyond the information that is available at the time. Instead, findings should be stated honestly and the need for a referral should be outlined. Offering guesses or percentages should be avoided.
- The finding of a condition should not entirely dominate the scan. For example, if the baby’s gender would normally be identified at this scan, this should still be done, should parents wish.

## Ending the scan

While it may not be possible to reduce parental shock and distress within the time-frame of a scan, the following steps can help support expectant parents with the subsequent stages of their journey.

### Avoid positive reframes

When expectant parents are distressed, it can be a natural tendency to try and comfort them by highlighting the positives in their situation. However, positive reframes at this point are likely to be perceived as invalidating and should be avoided. For example, sonographers and ultrasound practitioners should not point out a pregnancy was early on, that they already have a child or that they can ‘try again’. If expectant parents have lost a twin, it should not be highlighted that they still have the other twin.

### Offer practical information

Any pertinent practical information should be offered. For example, it can be helpful to tell expectant parents who have suffered intra-uterine baby loss to expect the presence of passive movement.

### Pictures

Expectant parents should be offered a scan photo. If they do not want this, they should be informed that one will be saved in their file in case they want a copy at a later date.

### Written information

Any information that expectant parents will need to remember, such as contact numbers or names of departments, should be written down. It is also helpful to write down the names or details of any relevant support organisations they can contact. This simple act of ‘signposting’ has been associated with an overall more positive perception of care in some parent groups.^24^

### Directing the parents

Wherever possible, expectant parents should be guided out of the scan room (and out of the department, where necessary) via an exit which does not route them through the main waiting room. If they have to wait, a space should be found which is away from the main waiting room.

### Self-care

While communication is natural, putting a probe on a pregnant woman and identifying a complication or potential complication is not. Delivering news via ultrasound is a uniquely demanding situation which puts a strain on healthcare professionals. This should be recognised in the provision of emotional support to sonographers and ultrasound practitioners.

## Discussion

The current project aimed to develop the first consensus guidelines for the communication of unexpected news via obstetric ultrasound. The guidelines were designed to cover all aspects of obstetric scanning which may be undertaken by sonographers or ultrasound practitioners working in UK EPU and FASP settings and as such included recommendations for pregnancy and baby loss at all gestations and the initial discovery of foetal conditions. Contributors reached agreement for each recommendation provided, resulting in the development of detailed consensus guidelines.

The need to improve the delivery of unexpected news via obstetric ultrasound has long been recognised,^2–4,25^ but there have been few advances in this area. Lack of consensus regarding the specific words and phrases which sonographers should use has been one factor inhibiting improvement in this area. This lack of consensus may have arisen for two main reasons. First, sonographers and ultrasound practitioners can encounter a wide range of unexpected findings in their practice, from anembryonic miscarriage to uncertain indicators of a possible foetal condition. Deciding which terms are appropriate for this breadth of possible findings requires a wide range of expertise and is beyond the scope of any individual or single disciplinary group to resolve. Second, as the UK is one of the few countries where sonographers and ultrasound practitioners deliver unexpected news as standard, there is no international precedent which can be drawn on. The current project overcame these issues by drawing together interdisciplinary healthcare professionals, lay experts, third-sector organisations, policy experts and researchers to discuss, consider, problem solve and reach consensus on these issues. The resulting guidelines are the first of their type and as practitioners in other countries, such as Australia, are now considering adopting a policy of immediate news disclosure in obstetric ultrasound,^7,8^ they may have international impact in future.

These guidelines aim to support the improvement of patient-centred care by making best practice recommendations accessible to practitioners. However, it is also hoped that by reducing ambiguity regarding news delivery, they may help to reduce sonographers’ and ultrasound practitioners’ stress levels. Stress reduction interventions in this group are much needed; sonographers have elevated rates of burnout internationally,^26,27^ and a recent UK study found that a majority were reporting symptoms of burnout and possible minor psychiatric disorder.^17^ Furthermore, sonographer vacancy rates are high, both in the UK^15,28^ and internationally,^29^ with a deleterious impact on patient waiting lists.^28^ As such, any intervention which may reduce sonographer stress is pertinent and timely.

## Strengths and weaknesses

The consensus guidelines benefitted from the contribution of a wide range of professionals and experts representing all relevant stakeholder groups. They also benefited from being developed via a clear, transparent approach which ensured all contributors agreed with the final recommendations. The guidelines are currently limited by a lack of research evidence to support their effectiveness in practice. It should also be noted that NHS FASP is an English screening programme, and while the recommendations and findings are likely to apply to FASP Scotland and Wales, this is less clear. Furthermore, Northern Ireland does not currently offer a FASP.

## Implications for policy and future research

There is no evidence-based training intervention to support sonographers and ultrasound practitioners with delivering unexpected news via obstetric ultrasound.^30^ A recent study found that overall, sonographers perceived a benefit from receiving news delivery training, but indicated that training courses could be improved.^17^ However, improvement has been hindered by a lack of consensus regarding the words and behaviours which should be used during new delivery encounters. Indeed, it has been found that recommendations provided in training can be lost in practice, when trainees observe that other sonographers are not following the recommendations in which they were trained.^1^ By making recommendations clear, transparent and widely available, it is hoped that the current guidelines will provide a basis for the development of more effective obstetric news delivery training courses.

At present, the consensus guidelines reflect the shared knowledge of the contributors regarding best practice in news delivery. However, whether these guidelines have the potential to improve practice will need to be tested in subsequent research studies; while studies in physicians suggest news delivery interventions are effective,^30^ no study has tested a news delivery intervention in sonographers and ultrasound practitioners. The implementation of these guidelines will also need to be explored, as a lack of consideration regarding implementation reduces the impact of clinical guidelines.^31,32^

## Conclusion

The project aimed to develop the first consensus guidelines for the delivery of unexpected news in obstetric ultrasound. The resulting guidelines are available online as supplmental material and at https://doi.org/10.5518/100/24 and provide recommendations covering how to set up scans, behaviours, words and phrases to use during scans and steps to take when ending scans. Key recommendations include: the use of neutral language, such as ‘expected’ and unexpected’ instead of ‘normal’ and ‘abnormal’ and ‘chance’ instead of ‘risk’; the default use of the term ‘baby’, even at early gestations, unless expectant parents use another term; and the use of honest communication throughout exchanges, even where findings are uncertain.

## Supplemental Material

sj-pdf-1-ult-10.1177_1742271X20935911 - Supplemental material for UK consensus guidelines for the delivery of unexpected news in obstetric ultrasound: The ASCKS framework Supplemental material, sj-pdf-1-ult-10.1177_1742271X20935911 for UK consensus guidelines for the delivery of unexpected news in obstetric ultrasound: The ASCKS framework by Judith Johnson, Jane Arezina, Liz Tomlin, Siobhan Alt, Jon Arnold, Sarah Bailey, Hannah Beety, Ruth Bender-Atik, Louise Bryant, Jen Coates, Sam Collinge, Jo Fishburn, Jane Fisher, Jan Fowler, Tracey Glanville, Julian Hallett, Ailith Harley-Roberts, Gill Harrison, Karen Horwood, Catriona Hynes, Lindsay Kimm, Alison McGuinness, Lucy Potter, Liane Powell, Janelle Ramsay, Pieta Shakes, Roxanne Sicklen, Alexander Sims, Tomasina Stacey, Anushka Sumra, Samantha Thomas, Karen Todd, Jacquie Torrington, Rebecca Trueman, Lorraine Walsh, Katherine Watkins, Gill Yaz and Natasha K Hardicre in Ultrasound
