# Supplementary material for: UK consensus guidelines for the delivery of unexpected news in obstetric ultrasound: The ASCKS framework
Source: Ultrasound. 2020 Aug 4;28(4):235–45. doi: 10.1177/1742271X20935911 (PMC13637473; doi:10.1177/1742271X20935911)
Supplement: Supplementary file 1 [file sj-pdf-1-ult-10.1177_1742271X20935911.pdf]

# CONSENSUS GUIDELINES ON THE COMMUNICATION OF UNEXPECTED NEWS VIA ULTRASOUND

# Improving News Delivery in Ultrasound (INDira)

## Contributors

Siobhan Alt, Fetal Anomaly Screening Programme

Jane Arezina, University of Leeds

Jon Arnold, Tiny Tickers

Sarah Bailey, University Hospital Southampton

Hannah Beety, Independent Midwife

Ruth Bender-Atik, Miscarriage Association

Louise Bryant, University of Leeds

Jen Coates, SANDS (Stillbirth and Neonatal Death Society)

Sam Collinge, University Hospital Coventry and Warwickshire

Jo Fishburn, Northumbria Healthcare NHS Foundation Trust

Jane Fisher, Antenatal Results and Choices

Jan Fowler, SOFT (Support Organisation for Trisomy 13/18)

Tracey Glanville, Leeds Teaching Hospital NHS Foundation Trust

Julian Hallett, Down's Syndrome Association

Natasha Hardicre, Bradford Institute for Health Research

Ailith Harley-Roberts, Sunshine and Smiles

Gill Harrison, City, University of London

Karen Horwood, Independent Lay Expert

Catriona Hynes, Sheffield Hallam University

Judith Johnson, University of Leeds

Lindsay Kimm, Public Health England Screening

Alison McGuiness, Mid Yorkshire NHS Foundation Trust

Liane Powell, Fetal Anomaly Screening Programme

Lucy Potter, Leeds Maternity Voices Partnership

Janelle Ramsay, Independent Lay Expert

Roxanne Sicklen, Royal Free London NHS Foundation Turst

Pieta Shakes, Southern Cross University

Alex Sims, Manchester University NHS Foundation Trust

Tomasina Stacey, University of Huddersfield

Anushka Sumra, Birmingham City University

Samantha Thomas, University of Sydney

Karen Todd, Department of Health and Social Care

Liz Tomlin, Bradford Teaching Hospitals NHS Foundation Trust

Jacquie Torrington, City, University of London

Rebecca Trueman, Lay Expert, Miscarriage Association

Katie Watkins, Leeds Teaching Hospitals NHS Foundation Trust

Lorraine Walsh, Birmingham City University

Gill Yaz, SHINE (Spina Bifida and Hydrocephalus Information, Networking and Equality)

# Contents

Contributors ..... 1
Preface ..... 5
Basic Principles ..... 6
Compassion and Self-compassion ..... 6
Tone of Voice and Body Language ..... 7
Make no assumptions ..... 7
Involve the Partner ..... 7
Setting up the Scan ..... 9
Introduction ..... 9
Checking Understanding ..... 9
Assessing Feelings about the Pregnancy ..... 11
Checking Consent ..... 11
Explaining the Silence ..... 12
Sharing the Monitor ..... 13
Setting up the Scan in the First Trimester ..... 13
Checking their dates ..... 13
Explain if you think an internal scan may be needed ..... 14
When Expectant Parents Bring Children ..... 14
During the Scan ..... 16
Behaviours ..... 16
Neutrality ..... 16
'Baby'? ..... 17
Understand the Nature of Shock ..... 17
Expressing Regret ..... 18
Technical/Translation ..... 18
Reaffirm Emotions ..... 19
Challenging Issues ..... 20
When a trans-vaginal scan is needed ..... 20
Seeking a second opinion ..... 20
Managing uncertainty ..... 21
When things get off-track ..... 22
Phrases to Communicate Pregnancy Loss ..... 24
Pregnancy of Unknown Location ..... 25
Ectopic Pregnancy ..... 26
Molar Pregnancy ..... 26

- Early Loss Prior to a Heartbeat ........ 27
- Early Loss Following a Heartbeat ........ 28
- Diagnosis of Multiple Pregnancy ........ 28
- Loss of a Twin ........ 29
- Baby Loss ........ 29

Phrases to Communicate Fetal Conditions ........ 30

- Raised Nuchal Translucency ........ 31
- Spina Bifida ........ 31
- Normal Variants ........ 31
- Heart Conditions ........ 31
- Dysmelia ........ 32
- Dilated Lateral Ventricles ........ 32
- Suspected Agenesis of the Corpus Callosum ........ 32
- Life-limiting Conditions ........ 32
  - Lethal skeletal Dysplasia ........ 33
- Late Findings ........ 33

Managing Challenging Reactions ........ 34

- Lack of Understanding ........ 34
- Disbelief ........ 34
  - Acknowledge ........ 34
  - Monitor ........ 35
  - Second opinion ........ 35
- Anger ........ 35
- Silence ........ 35
- Crying ........ 36

Ending the scan ........ 37

- Avoiding Positive Reframes ........ 37
- Offer Practical Information ........ 37
- Pictures ........ 37
- Written Information ........ 37
- Directing the Expectant Parent/s ........ 38
- Self-care ........ 38

References ........ 39

Appendix 1: Further Sources of Information Regarding Consent ........ 40

Appendix 2: List of National third-Sector Organisations for Signposting ........ 41

Appendix 3: Further Information and Support for Sonographers and Ultrasound Practitioners ........ 42

Appendix 4: Checklist........................................................................................................................43

# Preface

There has long been an awareness that there is a need to improve the delivery of obstetric news via ultrasound, but there have been few advances in practice. I believe this is because improvement in this area is challenging; the answers are not obvious and do not reside within any one person or group. The current guidance document represents a collaborative endeavour towards tackling this challenge. It is designed for ultrasound practitioners and sonographers rather than lay audiences; however, it has been informed by a wide range of contributors, including health professionals, lay experts, public health and policy experts and representatives from third-sector organisations. As such, I believe it reflects the preferences and needs of women and their partners.

The recommendations outlined in this document were initially generated during a one-day workshop funded by the Society and College of Radiographers and the University of Leeds. This opportunity enabled discussion and offered a forum to reach initial consensus in relation to several recommendations. The initial document was drafted following this workshop and was then circulated to all authors for feedback. After two revisions, the guidance document was finalised.

The document explains some of the processes underlying news delivery in obstetric ultrasound – the 'psychology' of communicating in this setting. It also seeks to move beyond this, outlining specific behaviours and phrases which sonographers and ultrasound practitioners can use. This is because research indicates that this level of specificity is important to patients. In the scan room practitioners need to find particular words: they cannot communicate in principles (Tomlin et al., 2019). By providing these suggestions we hope to support sonographers and ultrasound practitioners on a practical level. These guidelines are not exhaustive; given the vast array of possible news delivery scenarios, this simply would not be possible. Instead we view this guidance document as an opportunity to make news delivery recommendations clear and transparent. We hope the recommendations we offer can subsequently be further improved via research and feedback. Similarly, the phrases offered here are not designed to be learned by rote. Instead, they are to be viewed as positive examples which can be adapted to fit with different sonographers' natural communication styles.

I want to highlight here that, in my view, obstetric ultrasound practitioners and sonographers in the UK are pioneering. In many other countries, they are not expected to deliver news and may even be proscribed from doing this. However, in the UK sonographers and ultrasound practitioners deliver news as standard. Research indicates that this practice is in line with expectant parents' preferences, although it may be a challenging task for practitioners (Jansson & Adolfsson, 2010; Larsson et al., 2010; Rådestad et al., 2014; Tomlin et al., 2019). As such, we want to recognise the efforts of the profession in the UK to improve patient care and the example they offer to other ultrasound practitioners and sonographers internationally.

Finally, I would like to comment on our use of the terms 'sonographers' and 'ultrasound practitioners'. Throughout the document we use both terms to reflect the difference in training and role between these groups. We regard 'sonographers' to be professionals who hold a Postgraduate Certificate or a Postgraduate Diploma in Medical or Clinical Ultrasound. 'Ultrasound practitioners' are a broader group who include nurses, midwives and medical professionals who have trained in specific aspects of ultrasonography. By making this distinction, I hope we can recognise the professional expertise of the individuals who deliver ultrasound scans in UK NHS hospitals.

*Judith Johnson, November 2019*

# Basic Principles

Delivering unexpected news via obstetric ultrasound is challenging, and the specifics of the interaction will vary significantly according to the finding which has been identified. However, this section will outline some 'basic principles' which are relevant to all news delivery scenarios in ultrasound.

## Compassion and Self-compassion

Compassion for the people you are speaking to is a foundation for better news delivery. Without compassion, accurate news delivery skill may still be ineffective, leaving expectant parents with a poor and distressing experience of care. With compassion, even the 'wrong' words can be forgiven, with expectant parents seeing past exactly what was said, to the well-meaning intentions of the health professional who spoke them.

Compassion can be understood as a concern for the suffering of others, coupled with a desire to ease this suffering (Neff, 2003). Feeling anxious or under threat is the antithesis of compassion; it narrows the focus of attention and thinking and inhibits emotional expression (Fredrickson, 2001). Essentially, feeling anxious stops us from being able to be compassionate. When delivering news, sonographers and ultrasound practitioners regularly experience a range of challenges which can elicit anxiety, including uncertain findings or rare, unfamiliar anomalies. By providing practical, evidence-based suggestions, our aim is that this guide can help to reduce sonographers and ultrasound practitioners' anxieties around news delivery and thereby help to facilitate compassionate care.

Alongside recognising the importance of compassion for women and their partners who receive unexpected news via ultrasound, it is necessary to consider the need to have compassion for yourself as an ultrasound practitioner in these situations. Delivering unexpected news is difficult in any setting; research suggests that people who receive news immediately begin to 'make sense' of this, trying to understand what it means for them and why it has happened (John et al., 2019). As part of this process, people tend to dislike the person who has conveyed the news to them even if, logically, they had no role in causing the event (John et al., 2019). This means that news delivery events are often experienced as negative interpersonal interactions, which can take a toll on professionals tasked with regularly conveying such news.

This challenge is amplified in ultrasound settings. Unlike other healthcare settings, sonographers and ultrasound practitioners have no time alone to plan how to deliver the news: the parents watch their face as they analyse the scan. Expectant parents may have no warning or reason to expect challenging news and may not recognise the real purpose of the scan in the first place. They are also unlikely to have a pre-existing relationship with the ultrasound practitioner. Together, these factors make ultrasound a uniquely difficult setting in which to deliver news. It is important that as a ultrasound practitioner, you recognise the constraints under which you are working and have compassion for yourself when you think you have said 'the wrong thing'. Given these challenges, it is impossible to always get it right, and beating yourself up for past mistakes will not help you to improve in future. Instead, accept that everyone in this situation would make mistakes sometimes; improvement is an on-going journey.

## Tone of Voice and Body Language

As discussed above, anxiety can inhibit emotional expression (Fredrickson, 2001). In fact all negative emotional states can have this impact, including stress and burnout. In sonography, anxiety or stress may lead to sonographers and ultrasound practitioners distancing themselves from expectant parents by quietly disengaging; falling silent, saying little and avoiding eye contact. It is also worth noting that sonography is a complex task which involves concentration on the monitor; the nature of the task alone may contribute to disengagement from women and their partners. We know disengagement is common because silence in the scan room is a frequently cited experience of many parents who receive unexpected news via ultrasound (Radestad et al., 2014).

It is important for sonographers and ultrasound practitioners to recognise that disengaging is a natural tendency for anyone in this situation, and to consciously counteract this by using a warm, gentle tone of voice and making eye contact. This is particularly important because in this guide, we will recommend the use of clear language in communication. For example, we recommend the use of the word ‘died’ when communicating news of intrauterine baby loss to expectant parents. Without warmth and engagement, such plain language will sound callous. With warmth, however, the use of clear terms acknowledges the reality of expectant parents’ experiences and can support open communication, reducing the risk that they will be confused about what has happened.

Alongside this, it is important to speak slowly. Again, anxiety tends to work against this by causing us to speed up, think more quickly and speak more quickly. Be aware of this tendency, pace your words and pause. This will increase the chance that the expectant parents you are speaking to will be able to absorb what you are saying.

## Make no assumptions

It is part of our hard-wiring as humans to draw on our own experiences and our observations of others’ behaviour to identify patterns. We use these patterns to help us predict the behaviours of others, which help us manage our social interactions. However, this natural tendency can be unhelpful when we meet people who do not conform to our expectations. In situations as sensitive as ‘news delivery’, these implicit expectations, or assumptions, can lead us to really ‘put our foot in it’. It’s important to hold an open mind when delivering news, remaining aware that people may not view the news in as positive or negative a way as you might expect them to based on their appearance, medical notes, or the ways you have seen other people react before them. This is particularly important to remember when communicating news of a fetal condition. Ultrasound sonographers and ultrasound practitioners’ interactions with expectant parents are brief and often lack context; this is a challenge to communication, but one that can be managed through the use of unassuming language. In this guide, we will make specific recommendations about phrases you can use which avoid value judgements. This section links with the section on ‘neutrality’ in the chapter entitled ‘During the scan’.

## Involve the Partner

Partners can often get overlooked in discussions during antenatal healthcare interactions. However, when a partner is present at the scan, it is important to consciously include them in your conversations from the beginning. By involving them, you reduce the likelihood that they will feel redundant from the experience and increase the likelihood that their shared memories of the interaction will be accurate. Following the receipt of unexpected news, expectant parents often go

into shock (Jansson & Adolfsson, 2010). When in shock, expectant parents are focused on making sense of the news; they are unlikely to accurately absorb and recall what is said later on. By making eye contact with both partners and speaking to both, you increase the chance that, as a couple, they will remember the information you communicate to them.

At the start of the scan, it can be useful to try and establish the nature of the relationship between the woman and her partner in order to inform how you communicate with them. Whilst asking directly about this may be inappropriate, introducing yourself and allowing a pause where the woman and her partner can introduce themselves can elicit this information in a non-directive and non-threatening manner, if expectant parents are comfortable providing this. It is also in line with the recommendations of the 'hellomynameis' campaign (hellomynameis.org.uk).

Sometimes partners can disengage following the delivery of unexpected news, which can be distressing for the pregnant woman. If this happens, focus your attention explicitly on the partner. Example phrases are:

'I notice you are very quiet. I know this news must be unexpected, what would be helpful to you right now?'

'This news must be unexpected for both of you, and must feel like a lot to take in. What questions do you have? I will do my best to answer them'

'Unexpected pregnancy news can be really difficult for both partners. Do you have any questions I can answer, or would you prefer a few minutes to take it in?'

Using this approach can help validate any emotions they are experiencing and help them to feel involved. Eliciting information from them can also help the next steps of the interaction to be more tailored to their needs.

# Setting up the Scan

Having a conversation prior to beginning the scan is important for building rapport with the expectant parents and setting their expectations. While women and their partners should have been provided with information about the nature and purpose of their scans at the time of their booking appointment, weeks or months may have passed since their appointment, and it is likely they will have forgotten some of what they were told. During this conversation, you should aim to:

- Introduce yourself and your role as a sonographer
- Check understanding of the purpose of the scan
- Assess feelings about the pregnancy
- Check consent
- Explain you will be silent at the start of the scan
- Explain how and when you will show them the monitor

In first-trimester scans, you should also:

- Check their dates
- Explain if you think an internal scan may be needed

## Introduction

Many expectant parents may not clearly understand the role of sonographers. A brief introduction, including your name, can help set their expectations and put them at ease. One phrase you may choose to use is:

> 'My name is [*insert name*] and I'm the sonographer that will be scanning you today. My job is to assess the health and development of your baby and provide a written report for the doctors and midwives to manage your pregnancy care. I will also tell you my findings at the end of the scan. The purpose of today's scan is...[*insert reason for scan*]'

## Checking Understanding

Check the woman's awareness and understanding of the purpose of the scan using the 'Teach Back' technique http://scottishhealthcouncil.org/patient__public_participation/participation_toolkit/teach-back.aspx#.XYjcB1VKjL4 . This has two principles. First, put the spotlight on yourself or the process rather than the patient, to reduce the likelihood that they will perceive this as a test or criticism. Second, you ask the expectant parent to tell you what they know about the information you have just given them. If it is a routine scan, possible phrases you could use include:

'I just like to check in before I start scanning so that I know we're both/all on the same page. Would you mind telling me what you understand the purpose of the scan to be today?'

'There's an awful lot of information provided to women during pregnancy. So I always like to check at the start of scans – could you tell me what you understand the purpose of this scan to be?'

'To be sure that I have explained everything clearly, could you explain to me what you understand the purpose of the scan to be?'

If the scan is not routine, but the woman has been referred to you by another professional due to concerning symptoms, example phrases you may consider using are:

'I just like to check in before I start scanning so that my understanding is right. Would you mind telling me what the midwife told you, about why they sent you?'

'We're a very busy centre and sometimes information isn't always passed on to us quickly. Would you mind telling me what you understand the purpose of the scan to be today?'

If the expectant parents don't seem to be clear on the purpose of the scan, this is an opportunity to gently provide the correct information. Reassure them that any misunderstanding is common. Affirm any correct information that they have and then add the additional information you think they need at this moment. Depending on the situation, possible phrases you could use include:

'The reason we are doing this scan is a little different. This scan will assess [*insert reason*]. But don't worry – it's common to be unsure. That's why I always ask at the start of the scan.'

'Yes, that's part of the reason we undertake scans. However, we will also be looking for [*insert reason*]'

If the referral has stated there may be pregnancy/baby loss and the expectant parents do not appear to understand, it is not necessary to correct their understanding at this point. However, this information will help inform the way you deliver the news, if your scan confirms that pregnancy/baby loss has occurred.

## Assessing Feelings about the Pregnancy

Asking a question about how the woman feels about her pregnancy serves several purposes. You can gather information around whether the pregnancy is wanted or unwanted, whether there have been any concerning pregnancy symptoms or whether the woman has any particular reasons to be anxious, such as a difficult obstetric history. For example:

> ‘Before I start the scan, I like to check in with women to see how they are feeling. So, can I ask, how are you feeling about the pregnancy?’

If the woman discloses any sensitive information in response to this question, use affirmative responses and reassurance, for example:

> ‘Thanks for sharing that with me. It helps me to be aware of your experience before I start scanning’

## Checking Consent

Checking consent before proceeding with the scan can help to communicate that 1) it is not mandatory to receive the scan and 2) that it is a serious medical assessment. Phrases you could use include:

> ‘Before I continue, I need to check – do you consent to proceeding with the scan?’
>
> ‘I like to check at this point, do you want to continue with the scan?’

It is important to be aware that all patients have the right to ask any questions related to their health and to be provided with the information that is available. They should also be offered the opportunity for screening at each scan and if this is declined you need to ensure that this is due to an accurate understanding of the screening test. Understand that consent is an iterative process; it cannot be assumed that decisions made in a previous appointment will still be the same at a subsequent appointment. For more information regarding consent in ultrasound, see guidance issued by the Society and College of Radiographers and the British Medical Ultrasound Society.

## Explaining the Silence

Many expectant parents have reported finding the silence during scanning an anxiety provoking time. They may not understand the concentration needed to interpret scan images and expect feedback immediately. Expectant parents who have previously received unexpected or challenging news via ultrasound may find silences particularly stressful. Explaining at the start of the scan that you will be silent and how long you will be silent for can help reduce this anxiety. Also explain at this time any reasons that may increase the length of the time you think you will need to be silent for, such as if you are new to scanning. For example:

'At the start of the scan I will then take some measurements which can be sometimes be difficult to get. To help my concentration I will be silent for a few minutes while I focus on the monitor. Don't let my silence worry you – this is a standard part of scanning'

'I think it has already been explained to you that I am a student. This will mean that I will need to be silent for a bit longer than you might have experienced in your last scan, as I need to concentrate particularly hard to make sure I measure everything correctly'

Some sonographers and ultrasound practitioners prefer to first report on whether a heartbeat is present prior to conducting the rest of the scan. For expectant parents who have experienced prior loss/es, this can help to ease their anxiety for the rest of the scan. Indeed, even in cases where a life-limiting condition is subsequently found, this doesn't negate the existence of the heartbeat and the fact that the baby is still living. If you choose to do this, you may want to consider using a phrase such as the following:

'I can see the baby's heart beating. I will now continue to do the rest of the scan, taking relevant measurements to check baby's health. I will be silent for a bit, while I do this'

However, in situations where you cannot find a heartbeat, informing expectant parents that you cannot see this and that this means that the baby may have died/they may have experienced a miscarriage could result in a shock reaction that prevents you from being able to complete the rest of the scan. In these situations, it is necessary to use clinical judgment to balance the potential distress that will be caused by extended silence with the need to gather measurements immediately. As a principle, it is important to minimise the length of time before you tell expectant parents what you have found. They will likely be aware that is something is not as expected from your behaviour and body language, and the longer it takes to receive confirmation of this, the longer their anxiety will have had time to build.

## Sharing the Monitor

Consider whether and how you will show expectant parents images on the screen. Expectant parents have previously reported finding it particularly difficult when sonographers and ultrasound practitioners start by showing them the screen, but turn this away from them if an unexpected finding is identified. A more cautious approach is to initially view the screen yourself, and then share this with expectant parents once you have undertaken an initial assessment. However, it's important to recognise that some expectant parents may not wish to see the screen. As such, phrase this as a question, for example:

> 'To begin, I will just have my own screen on. However, once I've finished my initial assessment, I can switch the other screen on, so you can see the scan images too. Would you like me to do this?'

If you identify pregnancy/baby loss or unexpected fetal development during the scan, do not presume that expectant parents will not want to see the screen. Instead, after you have communicated your initial findings, reiterate the offer. Possible phrases you could use include:

> 'Would you like me to talk you through the findings on the screen or would you prefer I just discuss them without the images?'
>
> 'Would you like to see? I can put the other screen on, to show you your baby?'

## Setting up the Scan in the First Trimester

### *Checking their dates*

If a woman is in her first trimester or this is her first scan, it is helpful to assess the gestation of their pregnancy and their confidence in this at the start. Asking this question after you have started the scan indirectly communicates that there is an unexpected finding and causes anxiety which can then have a negative impact on subsequent communication. Example phrases include:

> 'As this is your first trimester, I always like to check – when was your last period?'
>
> 'Can I also just check – how regular are your periods and how long is your cycle?'

*Explain if you think an internal scan may be needed*

Prepare expectant parents for what will happen if you are unable to gather the information you need from a trans-abdominal scan. You could consider using possible phrases such as:

> 'Sometimes I'm not able to see everything I need to at this first scan. If that happens, you can either empty your bladder and we can do an internal scan, or you can go home and wait a week to repeat the scan. If this happens, we can talk about which option you would prefer.'
>
> 'Sometimes I'm not able to see everything I need to when using the probe on your abdomen. If this happens, I can do an internal scan which can provide clearer images during early pregnancy. However, it is your choice, and if this is needed we can discuss this then'.

## When Expectant Parents Bring Children

Having children in the scan can be a source of distraction for sonographers and ultrasound practitioners, and if unexpected findings are identified, expectant parents may be less able to absorb the information you are communicating to them. For these reasons, as part of standard protocol, many hospitals ask expectant parents not to bring children to the scan. However, children are still often brought to scans for a variety of reasons. Sometimes expectant parents have not read the information they were sent properly; sometimes they directly contravene what they were asked as they would like their child to 'see' the baby. However, some expectant parents have no option – they may have no one else who can look after their child in order for them to attend the scan alone. Furthermore, different organisations have varying levels of support available in terms of professionals who can care for children while their parents are receiving a scan. Given the wide range of different possible situations, it is not possible to prescribe specific guidance which can be applied to every situation. Instead, it is important to remember that there are three principles which should be considered when making decisions:

1. Offering choice: Where possible, provide options about how to proceed with the scan, as this can help expectant parents feel more in control of a situation which they may feel they have little control over.
2. Avoiding delays in delivering news: Once unexpected news has been identified, it is important that this is communicated as soon as possible and certainly within the same day.
3. Duty of care: The underpinning principle must remain to provide the best care possible; prioritising expectant parent's needs and wellbeing.

One recommended approach is to allow children in the scan, but to explain that if an unexpected finding is identified, you will ask the partner or other adult accompanying the woman to leave the scan room with the child while you communicate this news. Alternatively, you may prefer to ask the woman to attend the scan alone, while the partner waits outside with the child.

A particular challenge is presented where the woman is not accompanied by another adult. In these scenarios, it is worth exploring why this is. For example, if this is due to a misunderstanding, it may be easier to rearrange the scan for another time when care can be arranged for the child. However, you may discover that the woman is unable to arrange childcare and is attending alone with the

child out of necessity. In this situation, if it is acceptable within the department you are working in, we recommend conducting the scan with the child present, as it could be the only way to ensure that the woman receives appropriate antenatal care.

If you do discover unexpected news when conducting a scan where a child is present, you may choose to ask for the woman's choice on how best to proceed. You could consider using the following phrases:

> 'I have found something unexpected on the scan. Would you prefer me to share this with you now, or would you prefer your partner to wait outside with your son/daughter while I talk with you about this?'
>
> 'I have found something unexpected on the scan. I can tell you more information now, but if you prefer, we could talk later today or make another appointment when someone is available to look after your son/daughter? Let me know which you would prefer, it's up to you.'

However, it is important to note that if you judge the news to be distressing, you may want to consider prioritising the 'duty of care' principle over providing choice or delivering the news immediately. The reality is that until the woman knows the news, she is not fully informed to decide whether she would want her child to hear this. In these situations, we recommend seeking another professional in your organisation who can look after the child while you communicate directly with the woman. It is important that the woman does not go home without having heard the news.

# During the Scan

## Behaviours

When delivering unexpected news to expectant parents, put the ultrasound probe down first, before turning to face them and making eye contact. They may not make eye contact back, and if this happens, continue to speak to them gently, acknowledging that they may be finding this interaction particularly challenging. Putting the probe down provides a behavioural cue and puts you in a physical stance which communicates engagement and care. Where expectant parents do not fluently speak English, this also provides an additional way to communicate that something unexpected has been found. Similarly, where expectant parents have hearing loss or use lip-reading to communicate, facing them directly increases the chance that they will be able to understand what you are saying.

When receiving news from an internal scan, some women prefer to be dressed and sitting in an upright position. Others prefer to see the screen in order to help them understand what has happened. For this reason, offer women the opportunity to get dressed before you talk with them. Present this question in an open manner, so that they do not sense you are steering them to make any particular choice. A possible phrase you could use is:

> 'I've seen something that I'm not expecting to see at this stage of pregnancy. Would you prefer to talk about this now while I can show you the screen, or would you prefer to get dressed, and then talk?'

Offering this choice allows women to choose what is more important to them in that situation – whether this is immediately hearing the news, or being dressed in order to have a conversation in a more comfortable position. It also gives them a degree of control over a situation that many people feel they have no control over.

## Neutrality

It is natural to project our own values onto the nature of the information we communicate to others. However, it is important to recognise that unexpected news in ultrasound is often not objectively 'good' or 'bad'. How it is received will depend on the situation, experiences and views of the expectant parents who are receiving the news. As such, it is important to avoid using phrases such as 'bad news' and 'good news' or 'normal' and 'abnormal' when describing findings. Instead, use neutral phrases such as 'expected' and 'unexpected'. It is also important to use the term 'chance' rather than 'risk' when conveying likelihoods of possible outcomes. This is consistent with the terminology used by Public Health England in their patient information leaflets. (https://www.gov.uk/government/publications/screening-tests-for-you-and-your-baby-description-in-brief). An example of how you could convey news of a possible miscarriage in neutral terms is:

> 'At this stage of pregnancy, I would expect to see that the baby has a heartbeat. At the moment, I can't see any sign of a heartbeat. It's possible that it may be too early to see the heartbeat today and we may be able to see this in the next week, but there's also a chance that this means the baby will not develop as expected and you might experience a miscarriage'.

To note here, while the term 'unexpected news' is usually preferable to non-neutral terms (e.g., 'bad news'), it should be considered that for a minority of parents, this news may not be unexpected due to their previous experiences.

## 'Baby'?

There has been an on-going debate about whether it is appropriate for sonographers and ultrasound practitioners to use the term 'baby' when delivering unexpected or challenging findings, or whether 'pregnancy' or 'fetus' are more appropriate terms. However, the current consensus within the INDIRA writing group is that the default term should be 'baby'. Most expectant parents prefer this term and will use it themselves. For these parents, any other term may be perceived as invalidating or cold. 'Baby' is also the term used in the literature provided by Public Health England (https://www.gov.uk/government/publications/screening-tests-for-you-and-your-baby-description-in-brief). However, listen to the language used by the woman you are scanning. If she or her partner use another term, such as 'pregnancy' or 'fetus' in their communications with you, switch to their preferred term.

## Understand the Nature of Shock

When expectant parents receive unexpected news via ultrasound, they may go into shock. The degree of shock will vary between people; some will experience a high degree of shock, whereas others who may have been pre-warned to expect the news may experience a lower degree of shock. The underlying process of shock is similar between people: they are assimilating new information into their broader understanding of themselves, their life and their world. Essentially, they are processing what this new information means for them. However, exactly how people initially react to this process varies widely according to the nature of the news and their personality, experiences and culture. Some people will be overtly emotional and expressive, crying and mourning. Others will be quiet, focused internally on their thoughts. Others still may go into denial, resisting the emotional distress that will come with digesting the new information.

The shock reaction begins when the expectant parent first realises that there is 'news'; this may be before the ultrasound practitioner or sonographer even speaks, in response to non-verbal cues. Shock has physical, cognitive, emotional, and behavioural dimensions. A consistent observation is that expectant parents may struggle to recall information which is provided to them during this period; they are mentally distracted by processing the meaning of the news. Understanding the nature of shock can help sonographers and ultrasound practitioners communicate better with expectant parents; most notably, it is important to understand that expectant parents will not want to be immediately presented with options and asked to make decisions. Some may ask for more information about the finding and it is important to respond to these questions honestly with the

information that is available to you at that time. Others may ask no further questions, preferring to focus only on the information that has already been offered to them. The offering of options and discussion around decisions should happen subsequently, outside the scan room.

## Expressing Regret

The issue of whether or not it is appropriate to say 'sorry' has been contentious. However, there is a consensus within the INDIRA writing group that it is appropriate to open the news by saying 'I'm sorry to tell you that….' in situations where there has been any form of pregnancy or baby loss, or where a life-limiting condition has been identified. In situations where a condition is identified which may not be life-limiting or is clearly not life-limiting, it is better to 'hold' the sorry. That is, deliver the news simply by saying 'I have found that...'. Wait for the expectant parents' reaction and then follow this up by saying, 'I'm sorry, I know this is not what you were expecting to hear'. The underlying principle here is that while the news of the baby's condition might be unexpected, you should not try to predict how this news will be received by the expectant parents. This is for two main reasons; first, where parents choose to continue with a pregnancy, they may recall an opening apology as a negative value judgment on their choice and on their baby, and it may cause them to feel hurt and angry. By 'holding' the sorry until after their initial reaction, you are instead apologising for any shock or distress they are experiencing, rather than the finding itself. Second, as soon as the utterance 'sorry' is delivered, it can cause parents to go into shock. As discussed above, this can hinder expectant parents' cognitive processing and ability to recall information. It is better to impart the key information immediately before expanding on this with the use of 'sorry'. It is crucial that a gentle, warm tone of voice is used when using this strategy (this is discussed further in the 'basic principles' section). An example of how you could use this strategy is below:

**Ultrasound practitioner**: 'I have found that I can't see your baby's arm below the elbow.'

**Expectant Parent**: 'What?! Are you sure?' [*becomes tearful*]

**Ultrasound practitioner**: 'I'm sorry, I know this isn't what you were expecting to hear, and I know this must be upsetting. I think it ends at the elbow, as the baby has moved while I have been scanning and I still cannot see its arm below this point.'

## Technical/Translation

When communicating with expectant parents, it is important to use lay terms to describe the finding, to help them understand what this means or could mean for them. However, it is also important to provide them with the technical term. This is because most expectant parents will search the internet for information about the results of the ultrasound scan. Without the correct technical term, it is more likely that they will come across information which is misleading or distressing before they find the information which will be most useful for them. Returning to the example above, after the practitioner might want to continue the interaction by then saying:

'When babies have a difference with their limbs, such as a shorter arm for example, the technical term for this is 'dysmelia'. This is a broad term, but it just means there is a difference with a baby's limbs'

As technical terms and their correct spellings may be unfamiliar to expectant parents, also provide these in written format for them to take away. It is best practice for all patients receiving antenatal scans (including early pregnancy) to receive a copy of their written scan report. Doing this can provide expectant parents with the technical terms. However, it is important to note that these should be verbally communicated prior being provided in the report. If expectant parents have not been familiarised with these terms verbally prior to reading them, they may find them surprising or confusing.

## Reaffirm Emotions

Reaffirming a person's emotions can help them to feel heard and understood. You can do this by 'naming' the emotion they seem to be expressing, whether this is sadness, shock, disbelief or anger and expressing regret for this. You can combine reaffirming statements with 'wish' statements, where you state, for example, that you 'wish you didn't have to say this', or you 'wish things were different' (Pollak et al., 2019).

If an expectant parent expresses an emotion and you don't acknowledge it right away, it is likely that the emotion will build and they will say it again with increasing emphasis until you do acknowledge it or the encounter ends (Pollak et al., 2019). Below is an example of how a sonographer or ultrasound practitioner could reaffirm a woman's emotions following a diagnosis of stillbirth. This example is drawn from one ultrasound practitioner's experience, and also includes a 'wish' statement:

**Expectant parent**: 'I don't believe you. Check again! You're just not doing it right. I don't believe you.'

**Ultrasound practitioner**: 'I'm so sorry, I know this must be shocking and really hard to take in. I wish things were different, but I'm afraid this is what I have found.'

It is important to use these statements in proportion with the expectant parents' displayed emotions: understating extreme distress by naming it as 'a bit upset' is likely to make expectant parents feel undermined. Conversely, overstating an uncertain shock reaction by naming it as 'devastated' could enhance parental distress. Similarly, where findings are uncertain or indicative of a fetal condition rather than a pregnancy loss, wish statements are not appropriate.

## Challenging Issues

### *When a trans-vaginal scan is needed*

Refer to trans-vaginal scans as 'internal scans'; some women may not know what 'trans-vaginal' means and the phrase 'internal' is more intuitive. When one of these is needed, provide the honest reason for this and recognise that some women would prefer not to have one of these, rather than know the information one could provide. Phrases you could use include:

> 'I can't see the baby on the abdominal scan. This could be because abdominal scans aren't very clear when baby is early in development. I would like to do an internal scan which should give a clearer view, is this ok?'

It is important that you offer the genuine reason that you need to conduct the internal scan; 'holding on' to the news until you have confirmed it is likely to cause building anxiety and could cause the expectant parents to feel misled. Some women will decline an internal scan for various reasons. If they decline, respect this choice and offer to re-scan them at a later date. Clarify their choice and what this will mean, for example:

> 'You have chosen not to have an internal scan. This means that I can't give you any more information today. However, we will scan you again in [*insert specific time frame*]. At that point, we would expect to be able to see baby on the abdominal scan. If we can't, it might mean that you have had a miscarriage.'

### *Seeking a second opinion*

You may need to involve a colleague in the scan to either confirm a finding you feel confident of, or to provide a second opinion on a finding you are uncertain about. If this happens, provide expectant parents with the honest reason that you are doing this. If possible, it is best to use the phone to call a colleague so that you can stay with the expectant parents. If you need to leave the room, give an indication of how long you will be. Minimise the length of time you are out of the room. Possible phrases you can use at this time include:

'I'm sorry, I can see a baby in the pregnancy sac but the baby's heart is not beating. At this point, I would expect to see a heartbeat so this means that sadly your baby has died. I am going to ask another sonographer to come in and scan you to confirm these findings; is that OK? I will be gone about 5 minutes.'

'I think I have found an unexpected finding on the scan; [describe in lay terms what you can see]. However, I'm not sure - it's possible that [insert a factor which is making you uncertain, such as the position of the baby or the resolution of the scan]. I'm sorry, but I need to get one of my colleagues who will also scan you. I will be as quick as I can - hopefully about 5 minutes.'

It is important that you do NOT offer an excuse such as 'Hang on, I just need to get someone else to help', or that you omit to introduce the person coming to give a second opinion altogether. Either of these behaviours may cause expectant parents to feel 'fobbed off' or anxious, and will negatively impact your interaction with them. You should never bring a colleague into the room without informing the couple first.

Where sonographers are working in hospitals without other obstetrically trained sonographers, the process of seeking a second opinion is particularly challenging, as this will involve an external referral. In this situation, the principles remain the same: it is important to be honest with expectant parents about why a second opinion is needed, and it is important to try and arrange for this to be provided as quickly as possible. However, in this sitaution it is important to consider the situation of the expectant parents  - do they have a car or will they be using public transport? Are they keen to receive a second opinion as quickly as possible, or does their situation mean that an appointment on another day might be more suitable? Bear these things in mind when helping to arrange their onward care.

### *Managing uncertainty*

When a finding is uncertain, it can be tempting to offer a 'best case' scenario or imply that the outcome will probably be positive. In the same way, it may seem most prudent to offer a 'worst case' scenario, in order not to give expectant parents false hope. Both approaches are an attempt to help expectant parents avoid uncertainty; uncertainty is intrinsically anxiety provoking and we are therefore motivated to avoid it, and to help others avoid it. However, when the initial prediction is inaccurate and other possibilities have not been outlined, expectant parents can feel let down or misled. A better approach is to initially outline all the possibilities, followed by your estimated outcome. Honesty should be a guiding principle in this process; attempting to protect expectant parents' feelings by softening reality could backfire further down the line. You may want to use a phrase such as:

'There are three possibilities, [*describe these in lay terms*]. I think the most likely possibility is that [*describe this possibility in lay terms*]'

You should aim to include the full breadth of possible outcomes, offering a balance between 'best case' and 'worst case' situations. Where you think there is a high level of uncertainty and don't feel able to offer any likely possibility, state this and reaffirm the expectant parent's emotions. If relevant, it may be helpful to acknowledge the limitations of ultrasound scans. You may also want to use 'wish' statements. Below are some example statements:

'I'm sorry, I know you want an answer today but at this point in time it's not possible to say anything for certain. We won't be able to tell you any more until the next scan in a week's time.'

'I'm sorry, but ultrasound scans are limited in what they can tell us, especially at this phase of pregnancy. At the moment, this is all I can see on the screen. I wish I could tell you more, but I have told you everything I know at the moment.'

'I know this is difficult, and I wish I could tell you more right now. But I have told you everything I can at this point.'

The key principles are to avoid catastrophising (an over-focus on possible negative outcomes) and to avoid offering false hope (suggesting 'everything will be fine' where it may not be). Instead, focus on honesty and balance.

### *When things get off-track*

Given the immediate nature with which it is necessary to communicate news in ultrasound settings, it is likely that sometimes, you may feel you have not said the 'right thing' straight away, and quickly regret this. If you think this has happened, there is nothing wrong with apologising and acknowledging your own limitations. While you cannot 'take back' what you have said, you can convey your positive intentions and your compassion for their situation. If you do this, however, be careful to place the focus on yourself and not the expectant parents or their situation, as this could serve to increase their anxiety. One phrase you could consider using is:

'I'm sorry, I don't think I delivered that information very well to begin with. Please forgive me, I sometimes struggle to get my words out in the best way'.

Do not point out that the finding is unusual, rare, or something you have not come across before as part of your apology. This will engender a sense of 'aloneness' or 'strangeness' in expectant parents and will increase their level of distress.

In some cases parents find it hard to understand what is being said. It is important to provide alternative explanations without making them feel inadequate. In this situation, one option is to say:

‘I’m sorry I am not explaining this very well for you… [*offer an alternatively worded explanation*]’

# Phrases to Communicate Pregnancy Loss

When communicating news of a pregnancy loss, it is important to remember that how distressing expectant parents find this news can depend on a range of factors, some of which may be unknown to you. Do not assume that early losses will be less distressing and do not minimise expectant parents' distress; acknowledge that they have lost a 'baby'. Below is a table of terms which should be avoided altogether due to their potentially hurtful or offensive nature, with alternative suggestions on the right:

| Terms to avoid altogether | Alternative terms | Example phrase |
|---|---|---|
| **Blighted ovum/ anembryonic pregnancy** | A baby which died very early on | 'I can see a pregnancy sac in your womb, which is the place where the baby grows. Sadly though, I cannot see a baby in the pregnancy sac as it is likely that it died very early on before we could see it on the scan' |
| **Products/Products of conception** | Tissue/Pregnancy tissue<br><br>Remains of the pregnancy | 'I can still see the tissue which was left in your uterus from when your baby was growing'<br><br>'There are still some remains of the pregnancy in your womb from when your baby was developing' |
| **Evacuation of retained products/Vacuumed out/Scraped** | Surgery to remove tissue from your womb | 'This is surgery to remove the tissue that was left in your womb from the developing baby' |
| **Non-viable** | Will not continue to develop | 'The baby will not continue to develop, and won't survive' |
| **Incompetent cervix/ Cervical insufficiency** | Opening before it should | 'Your cervix is 'dilating' – which means that it is opening before should' |
| **Abortion (or any variation of this, such as 'threatened', 'missed' or 'incomplete')** | Miscarriage | 'Unfortunately you have miscarried, but this is what we call a 'missed miscarriage', or a 'delayed miscarriage' which means that you are still carrying the baby but it has stopped developing' |
| **IUD/ Intra-uterine death** | Baby has died | 'I'm afraid I can see that your baby's heart has stopped beating [*pause*], and this means that your baby has died' |

Some terms are unlikely to be hurtful or offensive, but may be confusing. If you think it could be helpful for expectant parents to hear the technical term, for example, because you know they will subsequently see it on their notes or in order to facilitate their understanding and internet searching, be sure that you also provide the lay translation. Below is a table of some example phrases:

| Technical term | Lay translation | Example phrase |
|---|---|---|
| **ERPC/ Surgical management of miscarriage** | Surgery to remove tissue from the womb | 'This is surgical management of miscarriage, which is the technical term to describe surgery to remove what was left in your womb from the developing baby' |
| **Gestational/yolk/pregnancy sac** | The place in which the baby grows | 'I'm looking at the pregnancy sac, which is the place where the baby grows' |
| **Haematoma** | An area of bleeding in or near the pregnancy sac | "I can see there is an area of bleeding next to the pregnancy sac (the place where the baby grows). This is called a haematoma. " |
| **Fetal pole** | The first sign of a developing baby | 'I can see the first sign of a developing baby' |

Below, we offer specific suggestions for communicating different types of pregnancy loss.

## Pregnancy of Unknown Location

Do not use the acronym 'PUL' when communicating this finding. Instead, first describe what you are seeing, and then offer the technical term for this. Acknowledge that ultrasound is limited in what it can see. Also explain that since ectopic pregnancy cannot be ruled out, this will need to be monitored for. One example you may choose to use is:

> 'I have not been able to see your pregnancy on today's scan. We know that you are or have been pregnant as you have had a positive pregnancy test. We call these 'pregnancies of unknown location'. There are 3 possible outcomes; 1/ the pregnancy is very early and too small to see on the scan, 2/ the bleeding you have had has been a miscarriage, 3/ you have an ectopic pregnancy that I haven't been able to see. Ectopic pregnancies are where the baby is growing outside of the uterus. This is common in early pregnancy and we will need to do some more tests to help us work out which of these outcomes we are dealing with.'

Once the expectant parents have initially reacted to the news, reaffirm their emotions, using wish statements if you think this is helpful. Then, outline what will happen next. One example is provided below here:

> 'I'm sorry, I know this probably wasn't what you were expecting to hear and must be upsetting. I wish I could give you more information, but this is all the ultrasound can tell us at the moment. I will book you in for a follow up scan in two weeks. At this scan, we will be able to tell you more about how your baby is developing'

## Ectopic Pregnancy

First communicate what you have found in lay terms, followed by the translation and what this will mean for the baby. Open with the term, 'I'm sorry', as this will help communicate to the woman that you have found something serious. Also remember to acknowledge the loss of the baby; this can be overlooked in ectopic pregnancy situations due to the risk to the mother's health. One example is:

> 'I am sorry to say that the baby has implanted and started to grow outside your uterus. The technical term for this is an 'ectopic pregnancy'. Sadly, what this means is that your baby will not be able to develop and they won't survive'

Allow the expectant parents an opportunity to react to this news and to ask any questions. Some parents will ask whether the baby can be moved. If this happens, respond gently but honestly that it cannot. Next, acknowledge the health risks presented by the situation and emphasise your priority as a healthcare professional, which is to ensure the wellness of the woman. It can be helpful for women at this time to be reminded that they are important and you are focused on their care. You could consider using a phrase such as the following:

> 'Ectopic pregnancies can be dangerous for women. The most important thing is that we make sure you are ok. Because your care is our main priority, I need to refer you on to [*insert person/department*]'

Outline any actions you will take and clarify what will happen next in their care pathway.

## Molar Pregnancy

Describe what you have found in lay terms, followed by the technical term. Open with an expression of regret. For example:

> 'I'm sorry to say I have found something unexpected in the baby's development. What has happened is that the cells which should have become the placenta (afterbirth) have taken over the place where we would expect the baby to grow. The baby has not been able to develop and will not survive. The technical term for this is that we call it a 'Molar pregnancy'

Molar pregnancies carry a very small risk of cancer, and any expectant parent who searches on the internet will quickly discover this. As such, it is important that this is addressed and discussed before the woman leaves the hospital. However, presenting this in the initial news delivery discussion could

be overwhelming. If an expectant parent is already aware of this and asks questions, answer them honestly. However, it is otherwise better to ask the midwife or obstetrician who conducts the follow-up conversation with them to discuss this and provide reassurance.

### Early Loss Prior to a Heartbeat

While the pregnancy doesn't resemble a baby at this time, it's important to recognise that many expectant parents will not be aware of this and may be shocked by the use of technical language referring to 'gestation sacs', 'fetal poles', etc. If they have talked about their pregnancy with others, they will likely have been using every-day terms such as 'pregnant' and 'baby' to describe their experience. As such, it is best to default to these terms. However, if you notice that they are using other terms (e.g., 'embryo', 'fetus') switch to these instead. If your finding is uncertain, be honest about this. For example:

> 'At the moment, I can see a fetal pole, which is the first sign of a developing baby. However, at this point, I would also usually expect to see a heartbeat, and I can't see one. It is possible that it is too early to see one and we may be able to see this within the next week, but there's also a chance that that this may mean the baby won't develop any further, and may not survive'

Where a fetal pole is not visible (i.e., where the pregnancy is anembryonic) it is better not to use the term 'first sign of a developing baby' because if expectant parents ask to see the screen, this will not be visible. Instead, state clearly that there is a pregnancy sac but you cannot see a baby. Acknowledge however, that there was a baby and this has died. One possible phrase you may choose to use is:

> 'I can see a pregnancy sac in your womb, which is the place where the baby grows. Sadly though, I cannot see a baby in the pregnancy sac. What this means is that the baby died very early on before it was large enough to be seen on the scan'.

Give the expectant parents time to absorb this news, express their emotions and ask any initial questions. Respond to this by expressing regret if appropriate, reaffirming their emotions and outlining the next steps. For example:

> 'I'm sorry, I know this wasn't what you expected to hear, and it must be really upsetting. I wish I could give you more information at the moment, but honestly, this is all that can be seen at the moment. We will book you in for a follow-up scan next week. At the next appointment, we will be able to tell you with more certainty about how your pregnancy is progressing'

If the appointment is a follow up scan or you have another reason to be certain that the pregnancy will end in miscarriage (for example, if the CRL is ≥7mm and there is no heartbeat), offer this information in simple terms. You may want to offer technical terms (and their lay translation) if the expectant parents seem confused or keen for more information. For example:

**Ultrasound practitioner**: 'I'm sorry, the baby doesn't have a heartbeat. At this point, this tells us clearly that the baby won't develop any further and won't be able to survive'

**Expectant Parent**: 'But how can you be sure? It's so early'

**Ultrasound practitioner**: 'I can show you the screen and explain, if you'd like?'

**Expectant Parent**: 'Yes'

**Ultrasound practitioner**: 'So, you can see here the pregnancy sac – this is the place where the baby develops. In here is the fetal pole, which is the very first sign of a developing baby. But if you look carefully, there is no heartbeat. When the baby is this size, we would expect to see a heartbeat. Also, the baby hasn't grown since you were scanned last week. I'm sorry, but what this means is that the baby has died'

## Early Loss Following a Heartbeat

Communicate the news simply. If expectant parents ask questions about the cause, do not give a reason unless you are clear on this. Use wish statements if appropriate. Finish by clarifying the next steps. For example:

**Ultrasound practitioner**: 'I'm sorry to say that your baby's heart is no longer beating. This means that your baby will not continue to develop and will not survive'

**Expectant Parent**: 'But I had a scan last week and everything was fine! What happened? What caused this?'

**Ultrasound practitioner**: 'Scans aren't able to give us that information. I wish I could tell you, but honestly I just don't know. I know this must be shocking news and you will have a lot of questions right now. Do talk to the midwife/doctor about this - I am going to refer you to them now, and they will come to meet with you to discuss what has happened and what will happen next. I'm so sorry I couldn't give you better news'

## Diagnosis of Multiple Pregnancy

Finding a multiple pregnancy can be challenging, as you will not know how this news may affect expectant parents. Acknowledge that you have identified unexpected news and then communicate what you have found in simple terms. One possible phrase you may consider using us:

'I've seen something today during the scan and I'm not sure how you will feel about this. When I scan through I can see [two, three] babies' [*pause*].

## Loss of a Twin

When scanning twins, check both heartbeats are present before communicating with the expectant parents. If you notice that one twin's heart has stopped beating, do not try and do the second anomaly scan there and then. Instead, scan only for the information which is urgent before communicating the news. For example:

> 'I'm sorry to say that one of your baby's hearts has stopped beating, which means that this baby has died'

It is important you do not comment that the fact that the other twin is still alive is 'good news', as this is likely to perceived as dismissive and lacking in empathy. See section entitled 'Avoiding Positive Reframes' in the Chapter entitled 'Ending the Scan' for more information.

## Baby Loss

While the term 'I'm sorry' may be contentious in relation to some obstetric ultrasound findings, it is crucial that this is used in situations where ultrasound identifies a late pregnancy loss or stillbirth. This is also one situation where it is acceptable to use the term 'bad news'. For example, you may want to open your communication with 'I am afraid it is unexpected news…', or 'I'm sorry, I'm afraid I have found that….'. Do not use the term 'IUD' or 'Intra-uterine death'. For example:

> 'I'm sorry, I'm afraid I have found unexpected news. Your baby's heart has stopped beating, and this means that your baby has died'

It is likely that any expectant parent receiving this news will experience a high degree of shock and may not hear anything after your initial disclosure. Reaffirm their emotions and use wish statements in response to distress. Repeat any key information which they need to know several times, as they will struggle to retain information at this time. Note down any particular words, names or numbers which they may subsequently need. Respond to their questions honestly but do not be tempted to provide any information which you are not confident of; if this is later contradicted it will lead to confusion.

# Phrases to Communicate Fetal Conditions

When communicating fetal conditions, avoid comparisons to inanimate objects or vegetables/fruits, for example, DO NOT describe any part of baby's anatomy as being like a 'lemon' or 'banana' or 'rugby-ball'. Instead, use adjectives and shapes as descriptors, such as 'longer', 'shorter', 'more oval-shaped', etc. Some specific examples of words to avoid are in the table below.

| Terms to avoid altogether | Alternative terms | Example phrase |
|---|---|---|
| Normal | Expected | 'This looks as we would expect at this point in the baby's development' |
| Abnormal | Not as expected/unexpected | 'This is not what I would expect to see during this phase of baby's development' |
| Disorder | Condition | 'This means the baby might have a condition called [*insert technical name of condition*]' |
| Incompatible with life | Will not be able to survive | 'Unfortunately, this means that the baby will not be able to survive' |
| Lemon-shaped | Narrows | 'The baby's head narrows at this point, which may indicate something unexpected with baby's development' |
| Banana-shaped | Curved | 'This part of the brain is curved, which may indicate something unexpected with baby's development' |

The key principles of delivering news of anomalies identified via ultrasound are:

- Avoid value-laden language. The alternative terms above will help you to do this.
- Unless the condition is clearly life-limiting (e.g., anencephaly) or you are otherwise sure that it is appropriate to use in this scenario, hold the 'I'm sorry' until after you have delivered the news of the initial finding.
- Use both the technical term for the finding and provide the translation. Write down the technical term and give it to parents.
- Don't try to diagnose above your expertise, state honestly what you can see and the need for a referral. Avoid offering guesses or percentages.
- Do not let the finding of a condition entirely dominate the scan; if you would normally identify the baby's gender in this scan, still do this, should parents wish. Offer the expectant parents a photo; if they do not want this, let them know you will save one in their file in case they want a copy at a later date.

There are too many potential conditions to offer phrases for all of these here. However, we offer several possible findings and examples of how these might be communicated below. The exact phrases you will use will depend on what you can see, but the examples offered here draw on the principles outlined above.

## Raised Nuchal Translucency

'The fluid at the back of baby's neck, known as the nuchal translucency, is bigger than we would expect for this stage of baby's development. This might be a chance finding, but it might mean that your baby has a chromosome condition. There are further tests that we could carry out, if you wanted us to. We will arrange for you to see the screening midwife so she can talk to you about what this might mean and answer any questions you have.'

## Spina Bifida

'The baby's spinal cord (the big bundle of nerves running from the brain down the baby's back) or vertebrae (the bones of the spine) have not developed as they should and this is a condition known as spina bifida. You will need to get an expert opinion so we will refer you to the fetal medicine unit where one the specialists will talk to you about what this means for your baby's health'

## Normal Variants

'There are some small differences in the way that your baby has developed. This might just be a chance finding, but it might indicate that your baby has a condition which could impact on their health. We will refer you to the experts at the fetal medicine unit where they will talk to you about what this means for your baby'

## Heart Conditions

'I can see an issue with baby's heart. This could mean [*insert the 'best case' scenario*] but it could mean [*insert the 'worst case' scenario*]. I can't be sure right now and so I need to refer you to have a scan by a heart specialist to explain what that means.'

## Dysmelia

'I can't see your baby's hand below their wrist on their right side. I think that your baby's arm stops at the wrist on their right side'.

## Dilated Lateral Ventricles

'There are several spaces in the brain that contain fluid, these are called ventricles. Your baby's ventricles are bigger than what we would expect to see. There are different reasons why the ventricles may be bigger than expected, and these may or may not impact the health of your baby. We will refer you to the experts at the fetal medicine unit where they will be talk to you about more tests or what this may mean'.

## Suspected Agenesis of the Corpus Callosum

'There are some differences in the way your baby's brain has developed. Sometimes these differences happen when a part of the brain doesn't grow in the expected way and this may or may not impact the health of your baby. We will refer you to the experts at the fetal medicine unit where they will be talk to you about more tests or what this means for your baby'.

## Life-limiting Conditions

Unlike other conditions, it is appropriate to open with an expression of regret when communicating news of a life-limiting condition. However, be careful to make no assumptions; some expectant parents may choose to continue with a pregnancy with a low or absent chance of survival. One example of how you might communicate news of a life-limiting condition is presented below. However, it is important to note that you should only communicate this if you are absolutely certain about the life-limiting nature of the condition:

### *Lethal skeletal Dysplasia*

'Your baby's bones are very small. The technical term for this is 'skeletal dysplasia'. More commonly, this is called 'dwarfism'. I am sorry but your baby's chest is much smaller than we would expect for this stage of baby's development and the lungs will not be able to fully develop. This means that the baby will not be able to survive.'

## Late Findings

Occasionally, unexpected findings are identified on scans after the anomaly scan. Ultrasound sonographers and ultrasound practitioners report that these are challenging to deliver, because expectant parents are particularly unlikely to be expecting this news. Explain the finding in simple terms, similar with other unexpected fetal development findings. However, also explain why the finding may not have been identified on earlier scans and apologise for this. An apology is not necessarily an admittance of fault, but can be used to convey regret about the situation. An example phrase is:

'I have found something unexpected in the baby's development that we were not able to see at your previous scan. [*describe the specific finding you have identified*]. I'm sorry this wasn't identified on a previous scan, the reason for this could be that this has only developed later in your pregnancy. It could also be that it was present before, but we weren't able to see it then because your baby was smaller.'

# Managing Challenging Reactions

## Lack of Understanding

If you believe that a language barrier is causing the lack of understanding, contact an interpreter or follow your organisation's protocol. Similarly, if you believe that the lack of understanding is caused by hearing loss or deafness, ensure you are facing the woman (and her partner, if present) when you speak in case they use lip-reading. Consider contacting a sign-language interpreter, or following your organisation's protocol. If you have ruled out that a language or communication barrier is the main cause of a lack of understanding:

1. Ensure that you have used both the technical term for the finding and the lay explanation of this. If there is a common-use name for the finding, also use this. Write the technical term down on a piece of paper and give it to them. Provide sign-posting information to any relevant organisations.
2. Ask about their needs at this time. It is likely they are in shock – ask whether they need time to "take it all in" and ask what questions they have. They may not have any questions at that time, but asking an open question (e.g. 'what questions do you have?') invites opportunity to ask questions, more than a closed question (e.g. 'do you have any questions?')
3. When you refer to the screening midwife or relevant health professional, ensure that you inform them of the communication challenges you have experienced and the potential lack of understanding. Ask the professional to explore the expectant parents' understanding in more depth once their shock has had some time to subside.

## Disbelief

Disbelief or denial can be a coping response; a reaction to news which is so devastating that the expectant parent cannot bear at that time to absorb it. There are three main steps you can take when coping with an expectant parent demonstrating a disbelief reaction:

### *Acknowledge*

Acknowledge the shocking nature of the news and reaffirm their distressed emotions. Then, you may find it helpful to use a wish statement to reinforce your positive intentions and regret. Depending on the specific situation, possible phrases you could consider using are:

> 'I know this news is shocking, and it must be so sad to hear. I wish I had different/ better news for you, I'm so sorry that I don't.'
>
> 'I know this news must be really unexpected – not what you thought you would hear today, and may be hard to process.'

### *Monitor*

Ask the expectant parent if they would like to see the monitor. If they would, share this with them, and show them the finding. Describe the whole picture in addition to the specific finding. For example:

> 'Here is the baby's head, here is one of their hands, and you can see their foot here. Now, this is where their heart is. You can see the outline of baby's heart, but there's no movement. We would expect to see baby's heart beating, but it isn't, which sadly means that the baby has died.'

### *Second opinion*

Tell the expectant parents that you will seek a second opinion. Be clear on what you are doing, and why. For example:

> 'I will go and find another sonographer who can also look at the scan, to check my finding. I will be gone for 5 minutes while I find them".

## Anger

It is important to emphasise here that you have the right to feel safe at work; if an expectant parent ever makes you feel threatened, you do not need to tolerate this. Your safety, both psychologically and physically, is priority, and you should not tolerate abuse.

However, if an expectant parent expresses anger at a finding which you do not find threatening, the key technique to manage this is to acknowledge and reaffirm the distress underlying their anger and to reiterate your positive intentions towards them. If they feel heard and understood, it is more likely to diffuse their anger. If they do not feel heard, they may raise their voice or increase their intensity in an attempt to gain this reaction from you. Next, ask if there is anything you can do to help them; it is hard to be angry when a healthcare professional clearly means well and is motivated to assist you. One example response is:

> "I'm sorry, I know this isn't the news you want to hear. I know this is really upsetting and I wish I could tell you something different. Is there anything I can do at this moment that will be helpful? Do you have any questions, or would you like some time to absorb the news?"

## Silence

If expectant parents respond to the news with silence it can be unsettling; it may be unclear whether they have understood the news and it can be hard to know how they are feeling. If this happens,

view it as part of their shock reaction; know that some people prefer to take time to process the news quietly and prefer not to be given a lot of information at that time.

While silence prior to the news delivery should be minimised, sit patiently with silence afterwards. Silence and pauses are often helpful to parents but can be challenging for you. Try not to fill the silence to ease your own discomfort. When you know you will need to begin to close the session, ask the expectant parents about the questions they may have. Inform them of the next steps, and provide them with any relevant written information that you can. While silence does not necessarily indicate a lack of understanding, written information will ensure that they do have the correct information with them, should they need it.

## Crying

If expectant parents express a great deal of sadness or grief immediately, allow them time to experience and express these emotions. Do not try to keep talking with them through these moments; anything that you say at this time is unlikely to be heard or processed. You may find this situation awkward, but it is better to offer space and quietness than to try and intervene immediately. Once their sadness or grief seems to reduce, try and re-engage them by using a reaffirmation statement, before moving the discussion forwards.

# Ending the scan

## Avoiding Positive Reframes

A natural part of many people's coping mechanisms is to positively reframe challenging experiences. This is often called 'looking for the silver lining' and involves looking for the positive things which are present in negative events. While this is a helpful personal strategy, it can be very unhelpful to do this for other people. Avoid doing this for expectant parents who have recently received challenging news. Do not point out the pregnancy was early on, that they already have a child or that they can 'try again'. If they have lost a twin, do not point out that they still have the other twin. Any positive reframes at this point will be perceived as invalidating; a minimisation of their distress. Instead, focus on compassion and reaffirming their emotions. It may be tempting to try and 'fix' their distress by highlighting positives, but it is important to accept that distress is likely to be a natural part of their journey.

## Offer Practical Information

If you are aware of pertinent practical information that will benefit the expectant parents, do offer this. For example, it can be helpful to tell expectant parents who have suffered stillbirth to expect the presence of passive movement. Write down any information that you think they will need to remember, such as contact number or names of departments they will be in touch with. Always communicate 'next steps' so expectant parents can anticipate what will happen.

## Pictures

Do not assume that if an expectant parent has received news of unexpected fetal development or if their baby's heart has stopped beating that they will not want a picture. Instead, offer a picture. If they choose not to be given this, print one anyway for their file, and tell them it is there. Pictures can be an important tool for making memories. Parents who are in shock may reject a picture at the time but later regret this; saving one on their file means that they can still access this later if they change their mind.

## Written Information

Providing written information is beneficial for two main reasons. First, some parents may struggle to ask questions and prefer to take information in more slowly; providing written information means they can assimilate new knowledge at their own pace. Second, due to shock, many parents will forget information that is imparted to them verbally. Providing written information means they have an accurate source of knowledge they can refer back to. As noted in previous sections, it is important to write down any terminology which you think is relevant for them but may be less common or hard to spell.

Expectant parents also often appreciate being signposted to relevant organisations (see Appendix 2). Providing this information can reduce the risk that they will find inaccurate or upsetting information via internet searching. In research studies, expectant parents often report greatly appreciating being sign-posted to these organisations which can help them to understand the non-medical aspects of

their baby's condition. In one study, it was found that the simple act of sign-posting was linked with a more positive overall experience of care at this time (Johnson et al., 2016).

## Directing the Expectant Parent/s

Wherever possible, guide expectant parent/s out of the scan room (and out of the department, where necessary) via an exit which does not route them through the main waiting room. If they have to wait, find a space which is away from the main waiting room. Expectant parents who have to be surrounded by pregnant women with presumably uncomplicated pregnancies immediately after receiving unexpected news report finding this very upsetting. However, it still important to acknowledge that the moment they find themselves somewhere different to where they expected to be will be a tough one. Where appropriate, reaffirm their emotions during this time. Also be clear what will happen next and how long they can expect to wait before someone will come to speak to them.

## Self-care

Be kind to yourself. While communication is natural, putting a probe on a pregnant woman and identifying a complication or potential complication is not. Delivering news via ultrasound is a uniquely demanding situation which puts a strain on healthcare professionals. Forgive yourself for the times where you believe you said or did the wrong thing; know that there will always be situations which take you by surprise. Explore coping strategies which are useful to you, whether this is speaking to other local sonographers/ultrasound practitioners, friends outside of work or doing activities which you enjoy and take your mind off things. It is also important to be aware that several organisations offer information, advice and support directly to healthcare professionals as well as expectant parents. For a list of these, see Appendix 3.

# References

Fredrickson, B. L. (2001). The role of positive emotions in positive psychology: The broaden-and-build theory of positive emotions. American Psychologist, 56, 218.

Jansson, C. & Adolfsson, A. (2010). A Swedish study of midwives' and nurses' experiences when women are diagnosed with a missed miscarriage during a routine ultrasound scan. Sexual and Reproductive Healthcare, 1, 67.

John, L. K., Blunden, H., & Liu, H. (2019). Shooting the messenger. Journal of Experimental Psychology: General, 148, 644.

Johnson, J., Adams-Spink, G., Arndt, T., Wijeratne, D., Heyhoe, J., & Taylor, P. (2016). Providing family-centred care for rare diseases in maternity services: parent satisfaction and preferences when dysmelia is identified. Women and Birth, 29, e99.

Neff, K. D. (2003). The development and validation of a scale to measure self-compassion. Self and Identity, 2, 223.

Pollak, K. I. (2020). Teaching effective communication by modeling effective communication: Experiences of a communication coach for clinicians. Patient Education and Counselling, 103, 423.

Rådestad, I., Malm, M. C., Lindgren, H., Pettersson, K., & Larsson, L. L. F. (2014). Being alone in silence–Mothers' experiences upon confirmation of their baby's death in utero. Midwifery, 30, e91.

Tomlin, L., Parsons, M., Kumar, P. V., Arezina, J., Harrison, R., & Johnson, J. (2020). Learning how to deliver bad and challenging news: Exploring the experience of trainee sonographers–A qualitative study. Ultrasound, 28, 30.

# Appendix 1: Further Sources of Information Regarding Consent

British Medical Ultrasound Society (BMUS) and Society and College of Radiographers; Guidelines for professional ultrasound practice (revised annually). Current version:

https://www.bmus.org/static/uploads/resources/SCoR_BMUS__Guidelines_Amend_March_2019_final.pdf

Society and College of Radiographers:

https://www.sor.org/practice/obtaining-consent

https://www.sor.org/system/files/article/201801/consent_leaflet_print.pdf

Royal college of Radiologists:

https://www.rcr.ac.uk/publication/standards-patient-consent-particular-radiology-second-edition (currently under review)

Royal college of Obstetricians and Gynaecologists:

https://www.rcog.org.uk/globalassets/documents/guidelines/clinical-governance-advice/cga6.pdf

https://www.rcog.org.uk/en/guidelines-research-services/guidelines/clinical-governance-advice-6/

Royal college of Nursing:

https://www.rcn.org.uk/professional-development/publications/pub-006047

# Appendix 2: List of National third-Sector Organisations for Signposting

Antenatal Results and Choices: 0845 077 2290; 0207 713 7486; https://www.arc-uk.org

Down Syndrome Association: 0333 1212 300; info@downs-syndrome.org.uk; https://www.downs-syndrome.org.uk

Miscarriage Association; 01924 200799; info@miscarriageassociation.org.uk; https://miscarriageassociation.org.uk

Reach; 0845 130 6225; 020 3478 0100; reach@reach.org.uk; https://reach.org.uk/

SANDS; 0808 164 3332; helpline@sands.org.uk; https://www.sands.org.uk/

SHINE (Spina Bifida and Hydrocephalus); 01733 555988; firstcontact@shinecharity.org.uk; https://shinecharity.org.uk

SOFT; enquiries@soft.org.uk; https://www.soft.org.uk

Tiny Tickers; https://tinytickers.org

# Appendix 3: Further Information and Support for Sonographers and Ultrasound Practitioners

For further information regarding communicating news of pregnancy or baby loss, see the following online resources:

https://www.miscarriageassociation.org.uk/information/for-health-professionals/e-learning/

https://nbcpathway.org.uk/

For further information regarding appropriate terminology for communicating anomalies, the Fetal Anomaly Screening Programme (FASP) has a range of resources you can access online:

https://www.gov.uk/government/publications/screening-tests-for-you-and-your-baby-description-in-brief

https://www.gov.uk/government/collections/fetal-anomalies-screening-conditions-diagnosis-treatment

The following organisations are happy to provide support and advice directly to sonographers regarding news delivery practice:

Antenatal Results and Choices: 0845 077 2290; 0207 713 7486; https://www.arc-uk.org

Down Syndrome Association: +44 (0)333 1212 300; https://www.downs-syndrome.org.uk

SANDS; 0808 164 3332; https://www.sands.org.uk

# Appendix 4: Checklist

The following checklist summarises some of the key points to remember when delivering news via ultrasound.

| Key Steps | Things to consider |
|---|---|
| **Avoid assumptions** | Remain aware that people may not react in the way you might expect them to. Use neutral terms (e.g., 'unexpected' rather than 'abnormal') and make no assumptions. |
| **Set up the scan** | Gathering information and setting expectations prior to all scans can facilitate better communication in those where unexpected findings are identified. Use the preamble prior to undertaking the scan to:<br>• **Introduce yourself** and your role<br>• **Check understanding** about the purpose of the scan<br>• **Assess feelings** about the pregnancy<br>• **Explain that you will be silent** at times during the scan<br>• **Check consent** for the scan and any screening<br>• **Explain when and how you will show them the monitor**<br>• In the first trimester, also **check their pregnancy dates** and **explain if you think an internal scan might be needed** |
| **Clear, honest information** | Providing expectant parents with clear, honest information can help them to understand and process the news. The following suggestions can support clear communication:<br>• **Put down the probe, turn and make eye contact** before verbally communicating the news<br>• Use **technical terms but also provide the 'lay translation'** of what these mean<br>• **Communicate exactly what will happen next and why** – whether this is an internal scan or leaving the room to seek a second opinion<br>• Wherever possible, **offer written information and signpost** to relevant organisations which can offer further information and/or support |
| **Kindness** | Kindness and compassion are key to better news delivery. The following suggestions can support kindness in news delivery situations:<br>• Unless you hear expectant parents using other terminology (e.g., 'fetus'), **use the term 'baby' as a default**, even in very early pregnancy<br>• **Understand the nature of shock**: it is a common phase where parents 'make sense' of the news they have been told. People struggle to assimilate new information during this time; **avoid asking them to immediately make decisions** around pregnancy management.<br>• **Express regret**: Where a condition has been found**, hold the 'I'm sorry'** until after you have delivered the initial news and only if this feels appropriate. In doing this, you are expressing regret about their distress, not the finding itself. |
| **Self-care** | Remember that delivering unexpected news via ultrasound is uniquely demanding. Have compassion for yourself, as well as those you are scanning. Explore coping strategies which are useful to you and make time to care for yourself. |
